# What can we learn from 68 000 clinical frailty scale scores? Evaluating the utility of frailty assessment in emergency departments

**DOI:** 10.1093/ageing/afaf093

**Published:** 2025-04-20

**Authors:** Hugh Logan Ellis, Liam Dunnell, Ruth Eyres, Julie Whitney, Cara Jennings, Dan Wilson, Jane Tippett, Dan F Stein, James Teo, Zina Ibrahim, Kenneth Rockwood

**Affiliations:** King’s College London, Department of Biostatistics & Health Informatics, Social Genetic and Developmental Psychiatry Centre, Memory Lane, Southwark, London, SE5 8AF, UK; Dalhousie University Ringgold Standard Institution,Department of Medicine, Suite 1421-5955, Veterans' Memorial Lane, Halifax, Nova Scotia, B3H 4R2, Canada; University Hospital Lewisham, Lewisham, London, UK; Princess Royal University Hospital, Department of Clinical Gerontology, Orpington, Bromley, Kent, UK; King's College London, The School of Life Course & Population Sciences, Southwark, London, UK; King's College Hospital NHS Foundation Trust, Emergency Department, Lambeth, London, UK; Kings College Hospital NHS Foundation Trust, Department of Clinical Gerontology, Lambeth, London, UK; King's College Hospital NHS Foundation Trust, Emergency Department, Lambeth, London, UK; King’s College London, Department of Biostatistics & Health Informatics, Social Genetic and Developmental Psychiatry Centre, Memory Lane, Southwark, London, SE5 8AF, UK; King's College Hospital NHS Foundation Trust, Neurology Department, Lambeth, London, UK; King’s College London, Department of Biostatistics & Health Informatics, Social Genetic and Developmental Psychiatry Centre, Memory Lane, Southwark, London, SE5 8AF, UK; Dalhousie University Ringgold Standard Institution,Department of Medicine, Suite 1421-5955, Veterans' Memorial Lane, Halifax, Nova Scotia, B3H 4R2, Canada

**Keywords:** clinical frailty scale (CFS), emergency department (ED), electronic health records (EHRs), frailty assessment, older people

## Abstract

**Background:**

Emergency departments (EDs) in England are under significant strain, with increasing attendances and extended wait times, affecting frail older adults. The clinical frailty scale (CFS) has been implemented as a tool to assess frailty in ED settings, but its reliability and predictive accuracy as a screening tool remain debated.

**Objective:**

To evaluate the use and variability of the CFS in EDs and its association with patient outcomes, including discharge rates, length of stay, readmission and mortality.

**Methods:**

A retrospective cohort study of ED attendances at two London (UK) hospitals from 2017 to 2021. Data included CFS scores, demographics, clinical observations and outcomes. Comparative statistics, logistic regression, Cox proportional hazards models and competing risk regression were applied to examine CFS predictive validity.

**Results:**

In a sample of 123 324 ED visits, CFS scores strongly correlated with adverse outcomes: e.g. for long-term mortality (*n* = 33 475, events = 8871), each CFS single-point increase was associated with a 25% increase in mortality risk (95% CI 1.23–1.26). CFS scores varied significantly between raters and across visits, median difference two levels (interquartile range 1–3). Intraclass correlation coefficient analysis showed that 33.1% of CFS score differences was attributable to between-patient differences, 15.4% to inter-rater differences, with 51.5% residual variance from non-frailty factors, such as acute illness severity.

**Conclusion:**

The CFS is associated with crucial patient outcomes in the ED. Inter-rater variability and potentially confounding factors can limit its consistency. Automation to enhance CFS score reliability should be explored as a means to support proactive management.

## Key Points

- In 68 067 emergency department (ED) visits, clinical frailty scale (CFS) scores predicted admission rates, length of stay, readmission risk and mortality.
- CFS scores showed significant variability between raters and across visits for the same patient, suggesting potential reliability issues in busy ED settings.
- Acute illness severity markers (National Early Warning Score, laboratory tests) were associated with CFS scores, suggesting potential confounding of baseline frailty assessment by acute illness.

## Introduction

Emergency departments (EDs) in England are facing unprecedented pressures. In 2023–24, A&E attendances rose by 3.8% to 26.3 million, with only 72.1% of patients seen within the target 4-h window—well below the 95% standard [1]. Notably, 7.1% of attendances involved waits exceeding 12 h, highlighting the urgent need for better patient flow and resource allocation. In 2023 almost a quarter (24.2%) of those waiting 12 h in the ED (65% of whom were awaiting admission) ended up waiting 24 h or more, with an estimated 14 000 associated excess deaths relating to increased wait times [2].

A significant proportion of these ED attendances are patients living with frailty [3], who are particularly vulnerable to hospital-related harm and adverse outcomes [4]—this extends beyond the UK and is a global challenge [5]. Frailty is not inevitable with ageing and plans to slow its progression should be a priority for healthcare providers and commissioners [6]. Hospital attendance, whether leading to admission or not, represents a valuable opportunity to identify those living with frailty and initiate proactive assessment and management. How do we meet the needs of this patient population in the challenging environment of the ED?

In 2019, NHS England outlined that, within 30 min of arrival, to help guide clinical assessment and decisions on care pathways [7] all people over 65 years who present to the ED should receive a frailty assessment, such as the clinical frailty scale (CFS) [8] which has been suggested as the most appropriate [9]. However, the application and effectiveness of applying the CFS in the high-pressure, time-constrained environment of the ED remains unclear, and questions have been raised about reliability of its use in these settings. A recent scoping review by Fehlmann *et al*. [10] examined 34 studies on the use of the CFS in emergency medicine, revealing rapid growth in its use but inconsistencies in how it is applied and reported. Most studies employed the CFS as a predictor of adverse outcomes, commonly hospital admission and mortality.

There are concerns regarding the reliability of CFS assessments in the ED setting; time constraints, incomplete patient histories and high patient turnover can lead to variability in CFS implementation. Are the frailty measurements captured here truly accurate, or are they influenced by confounding factors, such as illness severity and inter-rater variability? Shrier *et al*. [11] found disparity between ED triage nurse and inpatient medical team assessments using the CFS, raising questions about the consistency of CFS scoring. Moreover, their study suggested that ED-assessed CFS scores had limited ability to predict outcomes like length of stay and hospital admission, challenging its validity in this context.

Conversely, other studies have shown more promising results. Munir Ehrlington *et al*. [12] demonstrated that patients with higher CFS scores in Swedish EDs had significantly higher admission rates (58% vs 36%) and longer ED stays (median 5 h 08 m vs 4 h 36 m). Similarly, Ng *et al*. [13] found that integrating frailty assessment into triage could identify patients at higher risk of admission or poor outcomes without increasing resource use. Elliott *et al*. [14] considered 26 454 patients with CFS scores attending the ED and found that higher CFS scores correlated with risk of hospital admission and mortality, with readmission rate increased up to a score of 6.

Recognising and assessing frailty is important to individually tailor clinical management and avoid adverse health outcomes [4]. As the NHS grapples with rising ED attendances and prolonged wait times, the question of how to integrate frailty assessment into emergency care becomes increasingly relevant. Could CFS scores offer practical insights for ED decision-makers? How reliable are these scores in the fast-paced ED environment? Do they only measure frailty or are they capturing something else? Given the current challenges in patient flow, might frailty assessments help categorise patients by risk of admission and estimate their length of stay? In consequence, our objectives were to:

1. Evaluate the characteristics of patients with recorded CFS scores compared to ED patients aged over 70 without a CFS.
2. Assess the association between CFS scores and ED-relevant outcomes, despite potential inconsistencies.
3. Investigate the relationship between CFS scores and mortality (short-term and long-term).
4. Explore factors contributing to the variability in CFS scores completed in the ED.

## Methods

### Study design and setting

We conducted a retrospective cohort study of electronic health records (EHRs) from ED attendances between July 2017 and December 2021 from two hospitals in South London (UK). King’s College Hospital (KCH) is an inner-city tertiary centre with major trauma services, while Princess Royal University Hospital (PRUH) is a suburban district general hospital. CFS scoring began at KCH in July 2017 and PRUH in February 2018; we excluded PRUH records before this date to ensure consistency in data availability.

This observational study is reported following the STROBE (Strengthening the Reporting of Observational Studies in Epidemiology) guidelines [15]. A completed STROBE checklist is available in Supplementary Data.

### Data collection and processing

Using the CogStack [16] ecosystem for EHR processing, we extracted ED attendance data including CFS scores, demographics, clinical observations, laboratory results and outcomes for all patients over 70 within the study period. We excluded patients with indeterminate gender and those without attendance records (70 patients in total). For quality assurance, we performed standard data validation including range and consistency checks.

The CFS assessment was intended to be completed for all ED patients aged 70 and above. Assessments were typically conducted by the triage nurse on arrival, though for patients in resuscitation areas or urgent care who bypassed initial triage, the CFS could be completed later as a separate nursing assessment form. ED nurses were trained to estimate the patient’s baseline frailty status, focusing on their functional status 2 weeks prior to arrival.

Due to preliminary analysis showing disproportionately more CFS 1 classifications and fewer CFS 2 compared with population estimates, we combined CFS classifications 1 and 2 into a single category (CFS 1.5), having found no significant survival difference between these categories. We consolidated discharge destinations into seven categories and grouped presenting complaints into domains, using ‘generalised weakness’ as the reference being the most frequent of the ‘general’ presenting complaints. We calculated a laboratory-based frailty index (FI-Lab) from routine blood tests using all available laboratory values with reference ranges that were pertinent within 24 h of admission, expanding on the approach previously described [17] and commonly used [18].

### Statistical analysis

Our analysis employed R version 4.1.2, using:

1. Comparative statistics (Chi-squared, *T*-tests) for patient characteristics
2. Logistic regression for admission outcomes
3. Cox proportional hazards models for length of stay and survival analysis
4. Competing risks regression for re-attendance analysis
5. Linear mixed-effects models for CFS score variability

For each major outcome analysis, we produced two models: a full dataset analysis including all available data with basic adjustments (age, gender, site) and a complete case analysis including only cases with complete data for all variables—adding National Early Warning Score (NEWS), FI-Lab, and socioeconomic status using Index of Multiple Deprivation (IMD) from postcode data. Lower IMD ranks indicate areas of higher deprivation. We scaled variables to allow effect size comparison.

To understand CFS score variability, we examined relationships with patient characteristics, illness severity (NEWS), laboratory markers (FI-Lab), presenting complaints and temporal factors. We calculated intraclass correlation coefficients (ICCs) to assess inter-rater reliability.

For mortality analyses, we used last admission for inpatient mortality (defined as death before or within 24 h of discharge) and first admission for long-term mortality. We considered *P* < .05 statistically significant, without correction for multiple comparisons.

### Ethical considerations

We obtained ethical approval from the King’s Electronic Patient Record Interface committee (approval ID 20230411B).

## Results

Our study included 123 324 ED visits from 54 075 unique patients aged 70 years and older. Of these, 68 067 (55.2%) visits had a recorded CFS score, while 55 257 (44.8%) did not. Table 1 presents the characteristics of visits with and without CFS scores recorded, showing small but significant differences between groups.

**Table 1:** Patient characteristics for ED visits with and without recorded CFS scores. A comparative overview of demographic and clinical characteristics between ED visits where CFS scores were documented and those without recorded scores. Some patients appear in both columns due to different ED attendances having different CFS completion status.

Characteristic	Overall	No CFS	With CFS
Total visits	123 324	55 257	68 067
Unique patients	54 075	39 228	33 488
Female	67 015 (54.3)	29 465 (53.3)	37 550 (55.2)
Presenting complaint			
General: Weakness	8672 (7.0)	3520 (6.4)	5152 (7.6)
Airway: Breathing (other)	333 (0.3)	168 (0.3)	165 (0.2)
Airway: Difficulty breathing	2740 (2.2)	1602 (2.9)	1138 (1.7)
Airway: Short of breath	13 634 (11.1)	7561 (13.7)	6073 (8.9)
Circulation: Chest (other)	1788 (1.4)	919 (1.7)	869 (1.3)
Circulation: Chest pain	8136 (6.6)	3087 (5.6)	5049 (7.4)
Circulation: Collapse/fainting	1947 (1.6)	800 (1.4)	1147 (1.7)
Circulation: Palpitations	1934 (1.6)	962 (1.7)	972 (1.4)
Environmental	240 (0.2)	123 (0.2)	117 (0.2)
Eye	1783 (1.4)	1330 (2.4)	453 (0.7)
Gastrointestinal (other)	5289 (4.3)	1870 (3.4)	3419 (5.0)
Gastrointestinal: Abdominal pain	6627 (5.4)	2293 (4.1)	4334 (6.4)
General: Minor/administrative	4369 (3.5)	2135 (3.9)	2234 (3.3)
Genitourinary (other)	5056 (4.1)	1738 (3.1)	3318 (4.9)
Genitourinary: Unable to pass urine	2360 (1.9)	775 (1.4)	1585 (2.3)
Head and neck	2121 (1.7)	1134 (2.1)	987 (1.5)
Neurological (other)	4825 (3.9)	2787 (5.0)	2038 (3.0)
Neurological: Confusion	3610 (2.9)	1415 (2.6)	2195 (3.2)
Neurological: Dizziness	2432 (2.0)	793 (1.4)	1639 (2.4)
Neurological: Falls/unsteady	10 225 (8.3)	2545 (4.6)	7680 (11.3)
Neurological: Headache	2075 (1.7)	757 (1.4)	1318 (1.9)
Neurological: Limb weakness	3042 (2.5)	1873 (3.4)	1169 (1.7)
Neurological: Speech disturbance	2219 (1.8)	1310 (2.4)	909 (1.3)
ObGyn	215 (0.2)	76 (0.1)	139 (0.2)
Other	2341 (1.9)	1283 (2.3)	1058 (1.6)
Psychosocial: Behaviour change	1218 (1.0)	561 (1.0)	657 (1.0)
Skin (Other)	1477 (1.2)	921 (1.7)	556 (0.8)
Skin: Localised swelling/redness	2523 (2.0)	1240 (2.2)	1283 (1.9)
Trauma: Musculoskeletal (other)	8196 (6.6)	4351 (7.9)	3845 (5.6)
Trauma: Head injury	3267 (2.6)	1072 (1.9)	2195 (3.2)
Trauma: Injury of lower limb	2606 (2.1)	1415 (2.6)	1191 (1.7)
Trauma: Pain in lower limb	4904 (4.0)	2275 (4.1)	2629 (3.9)
Unwell adult	1120 (0.9)	566 (1.0)	554 (0.8)
Ethnicity			
Asian	4779 (3.9)	2027 (3.7)	2752 (4.0)
Black	18 387 (14.9)	7181 (13.0)	11 206 (16.5)
Mixed	95 (0.1)	45 (0.1)	50 (0.1)
Not stated	14 808 (12.0)	6968 (12.6)	7840 (11.5)
Other	612 (0.5)	239 (0.4)	373 (0.5)
White	84 643 (68.6)	38 797 (70.2)	45 846 (67.4)
Destination after ED			
Admitted	69 251 (56.2)	31 281 (56.6)	37 970 (55.8)
Ambulatory	2623 (2.1)	970 (1.8)	1653 (2.4)
Died in dept.	461 (0.4)	386 (0.7)	75 (0.1)
GP	7390 (6.0)	3394 (6.1)	3996 (5.9)
Home	35 682 (28.9)	15 003 (27.2)	20 679 (30.4)
OPD	5198 (4.2)	2671 (4.8)	2527 (3.7)
Other	2719 (2.2)	1552 (2.8)	1167 (1.7)
PRUH	51 656 (41.9)	27 077 (49.0)	24 579 (36.1)
Not recorded as deceased by October 2023	82 731 (67.1)	34 829 (63.0)	47 902 (70.4)
Age (mean ± SD)	81.89 (7.85)	81.63 (8.52)	82.09 (7.25)
NEWS score (mean ± SD)	1.96 (1.32)	1.96 (1.32)	1.97 (1.31)
Adjusted score (mean ± SD)	4.05 (1.87)	NaN (NA)	4.05 (1.87)
Fi-Lab	0.34 (0.11)	NaN (NA)	0.34 (0.11)
IMD rank (mean ± SD)	16254.93 (8783.98)	17062.20 (8882.92)	15608.23 (8649.79)
IMD decile (mean ± SD)	5.44 (2.66)	5.68 (2.69)	5.24 (2.62)
Length of stay (LOS) (mean ± SD)	5.24 (11.39)	5.39 (11.62)	5.17 (11.28)

Patients with a recorded CFS score were more likely to be female (55.2% vs 53.2%, *P* < .001) and to present with falls or unsteadiness (11.3% vs 4.6%, *P* < .001) or chest pain (7.4% vs 5.6%, *P* < .001). The CFS score completion rate differed significantly between sites, with PRUH recording CFS scores in only 36.1% of cases despite accounting for 41.9% of total visits.

### Clinical frailty scale score distribution and associated characteristics

As shown in Table 2, we observed significant trends across higher CFS scores in 68 067 ED visits. Higher CFS scores were associated with higher: age, admission rates, long-term mortality, NEWS scores, FI-Lab scores and longer hospital stays. Conversely, rate of discharge from ED and mean socioeconomic status decreased with higher CFS scores.

**Table 2:** Distribution of CFS scores and associated patient characteristics. This table illustrates the distribution of CFS scores among patients, including demographic and clinical variables. It also highlights the mean NEWS and FI-Lab scores and their association with CFS categories

Characteristic/CFS	Overall	1.5	3	4	5	6	7	8	9	*P*	Proportion of missing values
*n*	68 067	15 687	11 045	13 425	11 421	9211	5455	1524	299		
Unique patients	33 488	12 442	8707	9973	8395	6445	3989	1218	282		
Female	37 550 (55.2)	8310 (53.0)	5911 (53.5)	7288 (54.3)	6579 (57.6)	5337 (57.9)	3118 (57.2)	852 (55.9)	155 (51.8)	<.001	0
Ethnicity										<.001	
Asian	2752 (4.0)	571 (3.6)	535 (4.8)	574 (4.3)	469 (4.1)	331 (3.6)	203 (3.7)	54 (3.5)	15 (5.0)		
Black	11 206 (16.5)	1898 (12.1)	1965 (17.8)	2124 (15.8)	1918 (16.8)	1662 (18.0)	1153 (21.1)	413 (27.1)	73 (24.4)		
Mixed	50 (0.1)	10 (0.1)	10 (0.1)	16 (0.1)	7 (0.1)	6 (0.1)	1 (0.0)	0 (0.0)	0 (0.0)		
Not stated	7840 (11.5)	1972 (12.6)	1256 (11.4)	1559 (11.6)	1240 (10.9)	993 (10.8)	621 (11.4)	155 (10.2)	44 (14.7)		
Other	373 (0.5)	91 (0.6)	89 (0.8)	70 (0.5)	61 (0.5)	38 (0.4)	21 (0.4)	3 (0.2)	0 (0.0)		
White	45 846 (67.4)	11 145 (71.0)	7190 (65.1)	9082 (67.6)	7726 (67.6)	6181 (67.1)	3456 (63.4)	899 (59.0)	167 (55.9)		
Destination after ED										<.001	0
Admitted	37 970 (55.8)	6180 (39.4)	4843 (43.8)	7070 (52.7)	7499 (65.7)	6825 (74.1)	4137 (75.8)	1198 (78.6)	218 (72.9)		
Ambulatory	1653 (2.4)	660 (4.2)	366 (3.3)	376 (2.8)	167 (1.5)	65 (0.7)	15 (0.3)	3 (0.2)	1 (0.3)		
Died in dept.	75 (0.1)	10 (0.1)	7 (0.1)	6 (0.0)	11 (0.1)	11 (0.1)	19 (0.3)	4 (0.3)	7 (2.3)		
GP	3996 (5.9)	1193 (7.6)	971 (8.8)	838 (6.2)	496 (4.3)	287 (3.1)	161 (3.0)	42 (2.8)	8 (2.7)		
Home	20 679 (30.4)	6287 (40.1)	4068 (36.8)	4397 (32.8)	2823 (24.7)	1779 (19.3)	1019 (18.7)	251 (16.5)	55 (18.4)		
OPD	2527 (3.7)	958 (6.1)	575 (5.2)	529 (3.9)	271 (2.4)	137 (1.5)	46 (0.8)	9 (0.6)	2 (0.7)		
Other	1167 (1.7)	399 (2.5)	215 (1.9)	209 (1.6)	154 (1.3)	107 (1.2)	58 (1.1)	17 (1.1)	8 (2.7)		
PRUH	24 579 (36.1)	7918 (50.5)	3298 (29.9)	5309 (39.5)	3965 (34.7)	2626 (28.5)	1086 (19.9)	300 (19.7)	77 (25.8)	<.001	0
Not recorded as deceased by October 2023	47 902 (70.4)	13 114 (83.6)	8877 (80.4)	9998 (74.5)	7456 (65.3)	5127 (55.7)	2622 (48.1)	621 (40.7)	87 (29.1)	<.001	NA
Age (mean ± SD)	82.09 (7.25)	79.75 (7.06)	80.38 (6.59)	81.80 (6.85)	83.46 (6.98)	84.68 (7.11)	85.02 (7.41)	84.48 (7.41)	83.47 (7.71)	<.001	0
NEWS score (mean ± SD)	1.97 (1.31)	1.79 (1.15)	1.88 (1.25)	1.95 (1.31)	2.07 (1.36)	2.12 (1.43)	2.16 (1.46)	2.29 (1.50)	2.35 (1.48)	<.001	36.4
FI-Lab (mean (SD))	0.34 (0.11)	0.31 (0.11)	0.33 (0.10)	0.34 (0.11)	0.35 (0.11)	0.36 (0.11)	0.37 (0.11)	0.39 (0.11)	0.41 (0.13)	<.001	33.9
IMD rank (mean ± SD)	15 608 (8649.79)	17 714 (8836.18)	15 213 (8381.17)	15 913 (8789.96)	15 357 (8624.29)	14 314 (8267.96)	13 171 (7809.94)	12 960 (7681.39)	13 144 (8094.59)	<.001	2.8
IMD decile (mean ± SD)	5.24 (2.62)	5.88 (2.67)	5.12 (2.54)	5.34 (2.67)	5.16 (2.62)	4.85 (2.51)	4.49 (2.37)	4.44 (2.33)	4.53 (2.46)	<.001	2.8
Length of stay (LOS) (mean ± SD)	5.17 (11.28)	2.85 (7.86)	3.39 (9.18)	4.27 (9.77)	6.35 (11.98)	8.48 (14.64)	8.75 (14.73)	8.49 (14.70)	7.34 (12.05)	<.001	4.7

### Emergency department outcomes

In our complete case analysis (*n* = 28 535), each 1-point increase in the CFS score was associated with 12% lower odds of discharge to home (OR = 0.88, 95% CI 0.87–0.89, *P* < .001) and 13% lower odds of GP referral (OR = 0.87, 95% CI 0.86–0.88, *P* < .001). This relationship persisted after adjusting for illness severity and demographic factors (full models available in supplementary materials).

### Length of stay

**Figure 1:**
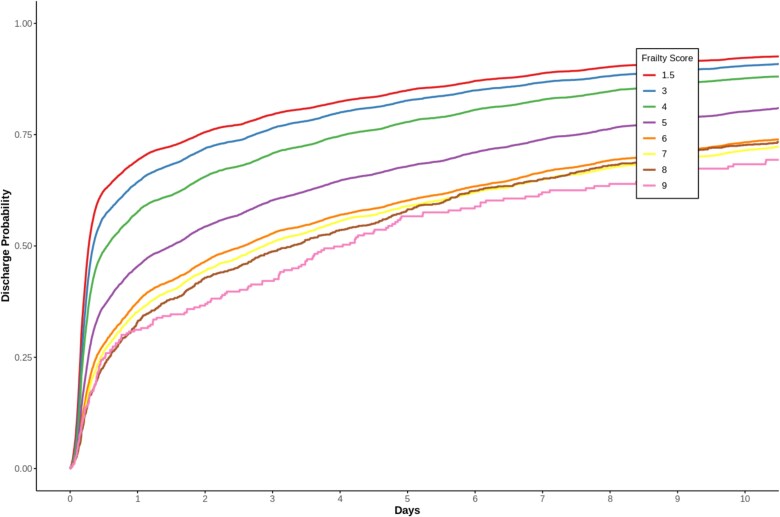
Kaplan–Meier survival curves illustrating the discharge probability over a 10-day period for various CFS scores. Each curve represents a different CFS score from 1.5 (very fit/fit) to 9 (terminally ill). The graph highlights a clear trend where the discharge probability decreases as frailty increases. Notably, the curves for CFS scores 6, 7, 8 and 9 closely overlap, indicating that beyond a certain level of frailty, further increases in the frailty score do not significantly affect the likelihood of discharge.

Each 1-point CFS score increase was associated with a 12% lower likelihood of discharge (HR = 0.88, 95% CI 0.88–0.89, *P* < .001) in complete case analysis (*n* = 27 489). When comparing standardised predictors, the CFS score showed the strongest effect on length of stay (HR = 0.79, 95% CI 0.78–0.80), exceeding both NEWS (HR = 0.89, 95% CI 0.88–0.90) and age (HR = 0.92, 95% CI 0.91–0.94). This relationship is illustrated in Figure 1, where Kaplan-Meier survival curves demonstrate a clear trend of decreasing discharge probability as frailty increases.

### Re-attendance

Using competing risk regression with death as a competing event (*n* = 33488), increasing CFS scores predicted higher re-attendance risk (HR = 1.03, 95% CI 1.02–1.04 per point). In analyses with standardised predictors, CFS scores remained the strongest clinical predictor of readmission (HR = 1.06, 95% CI 1.04–1.09), exceeding the effects of NEWS, FI-Lab, age and socioeconomic status.

### Mortality

**Figure 2:**
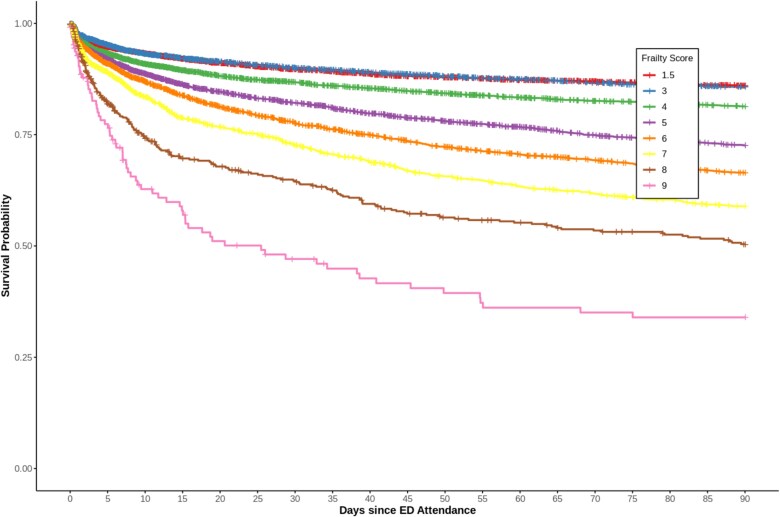
Kaplan–Meier survival curves illustrating survival probability over a 90-day period following ED attendance for various CFS scores. Each curve represents a different CFS score from 1.5 (very fit/fit) to 9 (terminally ill). The graph highlights a distinct trend in which the survival probability decreases as frailty increases.

CFS scores were strongly associated with both short- and long-term mortality. For long-term mortality (*n* = 33 475, events = 8871), each point increase in CFS score was associated with a 25% increase in mortality risk (HR = 1.25, 95% CI 1.23–1.26, *P* < .001). When standardised, the effect of CFS score (HR = 1.51, 95% CI 1.48–1.55, *P* < .001) exceeded that of age (HR = 1.37, 95% CI 1.34–1.40, *P* < .001). Inpatient mortality showed similar patterns, with higher CFS scores associated with greater mortality risk (HR = 1.18, 95% CI 1.14–1.23, *P* < .001). Figure 2 illustrates this relationship through Kaplan-Meier survival curves, showing lower 90-day survival probability with increasing CFS scores.

### Clinical frailty scale score variability

**Figure 3:**
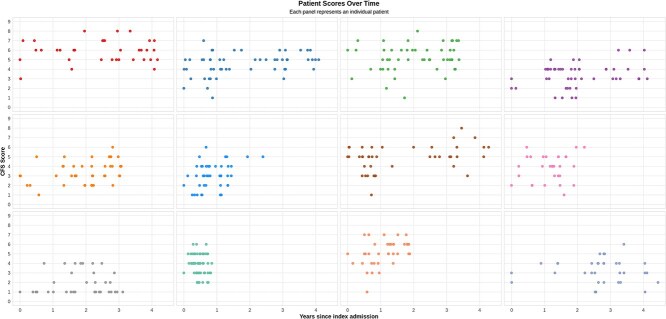
CFS scores over time for the 12 patients of 68 067 with the most frequent ED attendances. Each panel represents an individual patient. The *x*-axis shows years since their initial admission. The *y*-axis indicates the CFS score (range 1–9). Data points represent CFS scores recorded at each ED visit. This figure illustrates significant intra-patient variability in CFS scores over relatively short time periods. For example, the third panel in the first row (green dots) shows a patient scoring first 1(‘Very Fit’) and 8 (‘Very severely frail’) within ~3 months, and then surviving for at least 2 years with scores as low as 3 (‘Managing well’). This variability seems at odds with a CFS score that reflects a relatively stable measure of baseline frailty, highlighting potential inconsistencies in CFS assessment.

Figure 3 illustrates CFS score trajectories for the 12 patients with the most frequent ED attendances, revealing substantial intra-patient variability over short time periods.

The median range of CFS scores for patients with multiple visits (*n* = 33 488) was 2 points (interquartile range: 1–3), with ~0.2% of patients showing extreme fluctuations (40 of 7.5, 134 of 6.5, 21 of 6 and 474 of 5.5) between visits.

Linear mixed-effects models revealed significant associations between CFS scores and indicators of acute illness severity. Both NEWS (*β* = 0.12, *P* < .001) and FI-Lab (*β* = 0.15, *P* < .001) showed positive associations with CFS scores.

Presenting complaints were significantly correlated with CFS scores. Compared to ‘generalised weakness’, difficulty breathing was associated with higher CFS scores (*β* = 0.11, 95% CI: 0.01 to 0.21, *P* = .026). Confusion (*β* = 0.005, 95% CI: −0.07 to 0.08, *P* = .90) and falls/unsteady on feet (*β* = −0.07, 95% CI: −0.12 to −0.01, *P* = .015), while having slightly negative coefficients, were still scored higher relative to most other complaints. In contrast, chest pain (*β* = −0.70, 95% CI: −0.76 to −0.64, *P* < .001), eye problems (*β* = −0.74, 95% CI: −0.89 to −0.59, *P* < .001) and abdominal pain (*β* = −0.46, 95% CI: −0.52 to −0.40, *P* < .001) were associated with significantly lower scores. Patients at PRUH had significantly lower scores in a complete case analysis that controlled for socioeconomic status, presenting complaint, NEWS, FL-lab and age (*β* = −0.79, 95% CI: −0.98 to −0.60, *P* < .001).

The ICC indicated that 33.1% of the variance in CFS scores was due to differences between patients using a random effects model that accounted for repeated measurements and multiple raters (identified by individual assessor records in the EHR system, with all available ratings included for patients seen by different assessors), while 15.4% was due to differences between raters. The remaining 51.5% represents residual variance, which includes within-patient variability, including change in clinical status, and other unexplained factors.

Our analyses found no significant effects of time of day, day of the week or time of year on CFS ratings.

### Supplemental materials

The R code used for all analyses and the full statistical models are available as supplemental materials. Due to patient confidentiality concerns, the raw data cannot be shared publicly. However, researchers interested in replicating or extending this work may contact the authors to discuss potential collaborations or data access arrangements, subject to appropriate ethical and governance approvals.

## Discussion

Our study of over 68 000 CFS scores, the largest such investigation to date, provides compelling evidence supporting current guidelines mandating CFS assessment in ED settings, while highlighting opportunities to improve its implementation. These findings can inform service delivery through understanding local casemix patterns and monitoring quality of implementation over time. Through analysis of CFS implementation and its correlation with patient outcomes, we identified four key findings:

1. CFS scores demonstrate strong correlations with critical patient outcomes both within the ED and throughout the broader patient journey
2. Specific demographic and clinical factors influence the likelihood of CFS score documentation
3. There exists notable inconsistency in inter-rater reliability of CFS scores
4. Acute illness severity and characteristic frailty presentations are strongly associated with higher CFS scores

Several limitations warrant consideration. While our focus on two London hospitals may limit generalisability, our findings align with similar studies conducted elsewhere [11, 14]. The retrospective nature of our study necessitated reliance on previously collected clinical data, introducing potential inconsistencies and bias. Additionally, without a reference criterion for ED CFS scores and being unable to control for confounding factors or establish causality should be noted when interpreting results. Our data were collected before the 2023 Commissioning for Quality and Innovation [19] that incentivised frailty screening in EDs.

Our research extends findings regarding frailty assessment accuracy in EDs [11] by demonstrating CFS score variability across a larger sample size and broader patient population, including non-admitted patients. The observed median range of 2 points in CFS scores can shift patient classification between ‘managing well’ (CFS 3) and ‘mildly frail’ (CFS 5), or between ‘mildly frail’ and ‘severely frail’ (CFS 7)—categories that warrant different clinical approaches [7].

Variability is particularly concerning for patients with unidentified/underestimated frailty, who can miss opportunities to access specialised frailty services and Comprehensive Geriatric Assessment [20]—interventions that could mitigate frailty progression [21]. The financial implications are important; every 1% reduction in community-dwelling individuals transitioning to frailty potentially saves £4.4 million in annual social care expenditures [22].

Despite the inter-rater variability, our study demonstrates robust correlations between CFS scores and multiple patient outcomes: Increased likelihood of admission (with reduced discharge rates), extended length of stay, higher re-attendance rates, and increased mortality (both admission-related and independent). These findings reinforce trends observed in studies using the CFS [12–14, 23–27] and alternative frailty assessment tools [2, 9, 28, 29]. Our larger sample size adds statistical power to these observations, representing the most comprehensive study to date examining CFS scoring accuracy and its relationship to patient outcomes. Recent evidence from Karjalainen *et al*. further supports the value of systematic frailty screening in emergency settings—there targeted geriatric assessment following frailty screening significantly reduced both 72-h revisit rates (2.3% vs 4.6%, *P* = .001) and 30-day revisit rates (9.5% vs 15.7%, *P* < .001) compared to standard care. Early identification of frailty can enable targeted interventions can meaningfully improve patient outcomes and reduce ED attendances [30].

Our results open multiple avenues for improving frailty assessment and patient care in the ED. While the CFS proves an effective screening tool that correlates with patient outcomes, significant challenges remain in improving its accuracy and reliability. One promising approach involves implementing a ‘safety net’ system to identify frequent attenders with variable CFS scores, targeting these cases for more rigorous assessment. Additionally, understanding the demographic and clinical factors influencing CFS completion and scoring can inform targeted education programmes for staff undertaking assessments. This is exemplified by the higher CFS completion rates at KCH, where established Frailty Practitioners in the ED meant nurses could directly observe how their scoring led to rapid specialist frailty team involvement and improved patient care pathways.

Automation represents a particularly promising direction for future development. The observed correlations between NEWS, FI-Lab and CFS scores, combined with previous research on FI-Lab [17, 18], suggest the possibility of developing automated frailty assessment systems, perhaps using existing algorithms [31–33]. While predictive outcome models for frailty exist [34, 35], their reliance on manual frailty scores means they remain vulnerable to inter-rater variability. Data-driven automation could address this limitation while simultaneously reducing pressure on ED staff and resources.

Furthermore, readily available and accurate frailty measurements could facilitate more rapid decision-making and streamline patient care pathways. This potential extends into the prehospital setting, where early frailty identification could enable more appropriate deployment of specialist services. The London Ambulance Service conducts frailty scoring, but this information often remains disconnected from ED systems, with CFS scores typically only being documented for falls cases. Significant work is needed to develop effective, financially viable frailty measurement models and evaluate their impact on care delivery and ultimately, on patient preferences [36].

While frailty itself likely serves as a pathological driver, further research examining potential confounding factors for CFS-associated outcomes could identify interventions to improve patient care. Investigating how to effectively employ CFS-related outcome data could help tailor individualised care approaches, potentially reducing over-investigation and facilitating advance care planning to prevent unnecessary future hospital attendances. Automated frailty measurement could underpin these efforts.

The strong correlation between CFS scores and patient outcomes supports NHS England’s recommendation for universal frailty assessment within 30 min of arrival for patients over 65 presenting to acute services [7]. Likewise, it supports the NHS ‘Get It Right the First Time’ [37] recommendations for mandatory use of screening, developing an integrated approach to frailty that begins in the community and supporting the use of specialist interventions to prevent hospital-related harm, especially in people with CFS scores ≥6. Moving forward, emphasis should be placed on developing accurate, automated frailty measurement systems that could support clinical decision-making and reduce resource demands on already stretched ED services.

## Supplementary Material

aa-24-2567-File002

aa-24-2567-File003

## Data Availability

Due to patient confidentiality concerns, the raw data cannot be shared publicly. However, researchers interested in replicating or extending this work may contact the authors to discuss potential collaborations or data access arrangements, subject to appropriate ethical and governance approvals.
