# Supplementary material for: What can we learn from 68 000 clinical frailty scale scores? Evaluating the utility of frailty assessment in emergency departments
Source: Age Ageing. 2025 Apr 20;54(4):afaf093. doi: 10.1093/ageing/afaf093 (PMC12009543; doi:10.1093/ageing/afaf093)
Supplement: aa-24-2567-File002 [file aa-24-2567-file002.pdf]

Supplementary Materials for "What Can We Learn From 68,000 Clinical Frailty Scale Scores?"

Overview

This document contains the R code for the statistical analyses performed for our study, including data preprocessing, model development, and validation steps. The analyses are organized into several major sections as outlined below.

Table of Contents

Setup

Initial data preparation and library configuration. This section includes:

- Loading required R packages for statistical analysis, data manipulation, and visualization
- Importing raw data from Symphony electronic health record system
- Loading demographic data from PIMS
- Importing and processing laboratory-based frailty index results
- Loading and preparing observational data for both patient groups

Prep the Data

Data cleaning and transformation procedures including:

- Extraction and processing of NEWS scores from observational data
- Creation of derived variables and calculated fields
- Postcode data processing for IMD (Index of Multiple Deprivation) analysis

Tableone and Table Two

Generation of descriptive statistics and comparative analyses:

- Creation of summary tables for demographic and clinical characteristics
- Statistical comparisons between groups
- Analysis of key variables across different patient subgroups
- Presentation of primary outcome measures

Cox IP Deaths

Survival analysis focusing on inpatient mortality:

- Implementation of Cox proportional hazards models
- Analysis of mortality outcomes during hospital stay
- Adjustment for relevant covariates
- Model validation and diagnostics

Readmission Models

Analysis of hospital readmission patterns:

- Creation of readmission cohorts
- Time-to-readmission analysis
- Competing risks analysis for different outcomes
- Assessment of factors influencing readmission

Multiple Measurement

Analysis of repeated measurements and longitudinal data:

- Assessment of measurement variability over time
- Analysis of temporal patterns in clinical assessments
- Investigation of measurement reliability
- Evaluation of score changes over time

Random Effects Models

Mixed effects modeling :

- Implementation of random effects for patient and rater variability
- Analysis of temporal effects (seasonal, daily, time-of-day)

Each section contains detailed code, statistical outputs, and relevant diagnostics. Comments are included throughout in order to explain analytical decisions and interpretations.

Setup

In [22]:

```
#Setup
library(tidyverse)
library(lme4)
library(lubridate)
library(nnet)
library(broom)
library(car)
library(RColorBrewer)
library(viridis)
library(performance)
library(tableone)
library(survival)
library(survminer)
library(lme4)
library(broom.mixed)
library(knitr)
library(gt)
library(cmprsk)

options(repr.plot.width = 20, repr.plot.height = 10)
options(width = 220)

#Data - From Symphony
data2 <- read_csv('fscores_update.csv')
#Demographic data from PIMS
demo_data2 <- read_csv("demo_data2.csv")
# FI lab results calculated prior
fi_samples <- read_csv("fi_samples.csv") %>%
    rename(patient_TrustNumber = client_idcode) %>%
    mutate(time = ymd_hms(cfs_time),
           filab = 1 - filab) %>%
#Min number of tests for Filab
            filter(n > 5)
```

```r
#Observation Data from Symphony for both groups
obs1 <- read_csv('observations.csv') %>%
    select(obs = body_analysed, patientVisit_AttendanceNumber)

obsnocfs <- read_csv('observationsnocfs.csv') %>%
    select(obs = body_analysed, patientVisit_AttendanceNumber)

#Admission data for the same time for all comers to ED
everyone_ever <- read_csv('everyone_ever.csv')
day_1 <- min(data2$document_CreatedWhen)
unique_visits <- unique(data2$patientVisit_AttendanceNumber)

age_filter <- 70

everyone_afterday1_age <- everyone_ever %>% filter(document_CreatedWhen >= day_1,
                         patient_Age >= age_filter) %>%
mutate(PRUH = str_detect(patientVisit_AttendanceNumber, "PR"))

unique_visitsage <- unique(everyone_afterday1_age$patientVisit_AttendanceNumber)
#Postcode data for IMD
postcodes <- read_csv("postcodes4.csv") %>%
  mutate(across(starts_with("pcd"), ~ str_replace_all(., " ", "") %>% str_to_upper()))

imdlookup <- read_csv("imdlookup2.csv") %>% filter(`Indices of Deprivation` == 'a. Index of Multiple Deprivation (IMD)') %>% select( lsoa21[illegible]


# Get unique postcodes and clean them similarly
unique_postcodes <- unique(c(data2$patientAddress_PostalCode, everyone_afterday1_age$patientAddress_PostalCode)) %>%
  str_replace_all(" ", "") %>%
  str_to_upper()

# Filter and match the postcodes
slim_postcode <- postcodes %>%
  filter(pcds %in% unique_postcodes) %>%
  select(pcd = pcds, lsoa21cd)

postcode_ref <- imdlookup %>%

  right_join(slim_postcode) %>%
  select(clientaddress_postalcode = pcd, imd_rank, imd_decile)

postcode_ref_2 <- imdlookup %>%
  right_join(slim_postcode,) %>%
  select(patientAddress_PostalCode = pcd, imd_rank, imd_decile)
```

```r
#Add postcodes to data
data2 <- data2 %>%
mutate(patientAddress_PostalCode = str_replace_all(patientAddress_PostalCode, " ", "") %>% str_to_upper()) %>%
  left_join(postcode_ref_2, by = "patientAddress_PostalCode") %>%
mutate(PRUH = str_detect(patientVisit_AttendanceNumber, "PR"))
#Function to Extract NEWS scores from the obs
extract_news_components <- function(text) {
  # Define the components of interest
  components <- c("Date | Time", "Oxygen Saturation", "Resps (per min)",
                  "Blood Pressure (Systolic)", "Blood Pressure (Diastolic)",
                  "Pulse", "Temperature")

  # Replace \r\n with \n for consistency
  text <- gsub("\r\n", "\n", text)

  # Split the text into lines
  lines <- unlist(strsplit(text, "\n"))

  # Create a list to hold the key-value pairs
  data_list <- list()

  # Loop through the lines and extract key-value pairs
  i <- 1
  while (i <= length(lines)) {
    line <- trimws(lines[i])
    if (startsWith(line, "Nursing Observations > ")) {
      key <- trimws(gsub("Nursing Observations > ", "", line))
      if (key %in% components) {
        if ((i + 1) <= length(lines)) {
          value <- trimws(lines[i + 1])
          data_list[[key]] <- value
        }
      }
    }
    i <- i + 1
  }

  # Ensure all desired keys are present in the list
  for (comp in components) {
    if (!comp %in% names(data_list)) {
      data_list[[comp]] <- NA
    }
  }

  # Convert the list to a dataframe
  df <- as.data.frame(data_list, stringsAsFactors = FALSE)
```

```r
  return(df)
}

calculate_news_score <- function(row) {
  score <- 0

  # Respiratory Rate
  if (!is.na(row$RespRate)) {
    if (row$RespRate <= 8) {
      score <- score + 3
    } else if (row$RespRate >= 25) {
      score <- score + 3
    } else if (row$RespRate >= 21) {
      score <- score + 2
    } else if (row$RespRate >= 9) {
      score <- score + 1
    }
  }

  # Oxygen Saturation
  if (!is.na(row$O2Sat)) {
    if (row$O2Sat <= 91) {
      score <- score + 3
    } else if (row$O2Sat <= 93) {
      score <- score + 2
    } else if (row$O2Sat <= 95) {
      score <- score + 1
    }
  }

  # Systolic Blood Pressure
  if (!is.na(row$SysBP)) {
    if (row$SysBP <= 90) {
      score <- score + 3
    } else if (row$SysBP >= 220) {
      score <- score + 3
    } else if (row$SysBP <= 100) {
      score <- score + 2
    } else if (row$SysBP <= 110) {
      score <- score + 1
    }
  }

  # Pulse
  if (!is.na(row$PulseRate)) {
```

```
    if (row$PulseRate <= 40) {
      score <- score + 3
    } else if (row$PulseRate >= 131) {
      score <- score + 3
    } else if (row$PulseRate <= 50) {
      score <- score + 1
    } else if (row$PulseRate >= 91) {
      score <- score + 1
    } else if (row$PulseRate >= 111) {
      score <- score + 2
    }
  }

  # Temperature
  if (!is.na(row$Temp)) {
    if (row$Temp <= 35.0) {
      score <- score + 2
    } else if (row$Temp >= 39.1) {
      score <- score + 2
    } else if (row$Temp <= 36.0) {
      score <- score + 1
    } else if (row$Temp >= 38.1) {
      score <- score + 1
    }
  }

  # Level of consciousness is not in the given data, but we can assume "Alert" = 0 for conservative scoring
  # Adjust the row key here if you have a specific column for consciousness level
  # consciousness <- row["Consciousness Level"]
  # if (!is.na(consciousness) && consciousness != "Alert") {
  #   score <- score + 3
  # }

  return(score)
}
```

Prep the data

In [243…

```
process_news_data <- function(df) {
  df %>%
    rowwise() %>%
    mutate(parsed = list(extract_news_components(obs))) %>%
    unnest(cols = c(parsed)) %>%
    select(-obs,
           DateTime = 'Date...Time',
```

```r
            O2Sat = 'Oxygen.Saturation',
            RespRate = 'Resps..per.min.',
            SysBP = 'Blood.Pressure..Systolic.',
            DiaBP = 'Blood.Pressure..Diastolic.',
            PulseRate = Pulse,
            Temp = Temperature
    ) %>%
    mutate(
      DateTime = as.POSIXct(DateTime, format = "%Y-%m-%d %H:%M:%S"),
      O2Sat = as.numeric(O2Sat),
      RespRate = as.numeric(RespRate),
      SysBP = as.numeric(SysBP),
      DiaBP = as.numeric(DiaBP),
      PulseRate = as.numeric(PulseRate),
      Temp = as.numeric(Temp)
    ) %>%
    rowwise() %>%
    mutate(NEWS_Score = calculate_news_score(cur_data()),
           NA_Count = sum(is.na(across(everything())))) %>%
    ungroup() %>%
    group_by(patientVisit_AttendanceNumber) %>%
    arrange(NA_Count, DateTime) %>%
    slice(1) %>%
    ungroup()
}

# Apply the function to both dataframes
news_per_visit <- process_news_data(obs1)
news_per_visitnocfs <- process_news_data(obsnocfs)

news_per_visit <- process_news_data(obs1)

#Frailty scores are in string text format - need to be extracted
extract_last_8_chars <- function(text) {
  n <- nchar(text)
  if (n >= 8) {
    return(substr(text, n-7, n))
  } else {
    return(NA)  # Return NA if the text is shorter than 8 characters
  }
}



# Create a summary table to group presenting complaints
summary_table <- everyone_afterday1_age %>%
```

```
  group_by(patientVisit_RegistrationComplaint) %>%
  summarise(t_n = n()) %>%
  arrange(desc(t_n))
#Set the threshold
threshold <- 2500

#  Add the new column to the original dataset
everyone_afterday1_age <- everyone_afterday1_age %>%
  left_join(summary_table, by = "patientVisit_RegistrationComplaint") %>%
  mutate(presenting_complaint = ifelse(t_n >= threshold,
                                       patientVisit_RegistrationComplaint,
                                       ifelse(grepl(">>", patientVisit_RegistrationComplaint),
                                              sub(">>.*", "", patientVisit_RegistrationComplaint),
                                              "Other"))) %>%
  select(-t_n) %>%  # Remove the temporary 'n' column used for filtering
 mutate(presenting_complaint = as.factor(presenting_complaint)) %>%
 mutate(presenting_complaint = relevel(presenting_complaint, ref = 'General / minor / admin >> Generalised weakness'))# Convert to factor


#Process the patients with Frailty scores
scores <- data2 %>%
filter(document_CreatedWhen >= day_1) %>%
left_join(summary_table, by = "patientVisit_RegistrationComplaint") %>%
  mutate(presenting_complaint = ifelse(t_n >= threshold,
                                       patientVisit_RegistrationComplaint,
                                       ifelse(grepl(">>", patientVisit_RegistrationComplaint),
                                              sub(">>.*", "", patientVisit_RegistrationComplaint),
                                              "Other"))) %>%
  select(-t_n) %>%  # Remove the temporary 'n' column used for filtering
 mutate(presenting_complaint = as.factor(presenting_complaint)) %>%
 mutate(presenting_complaint = relevel(presenting_complaint, ref = 'General / minor / admin >> Generalised weakness'))%>% # Convert to fact
  mutate(
    last_8 = extract_last_8_chars(body_analysed),
    Frailty_score = if_else(str_detect(last_8, "Score \\d"), as.numeric(str_extract(last_8, "\\d")), NA_real_),
    score = as.numeric(if_else(str_detect(last_8, "Score"), str_sub(last_8, -1, -1), NA_character_)),
    rater = factor(document_TouchedBy),  # Convert rater information to a factor
    dest = case_when(str_detect(patientVisit_DischargeOutcome, "Admit") ~ "Admitted", #Summarise destinations
                     str_detect(patientVisit_DischargeOutcome, "Discharge") ~ "Home",
                     str_detect(patientVisit_DischargeOutcome, "Home|Left") ~ "Home",
                     str_detect(patientVisit_DischargeOutcome, "OPD|Clinic") ~ "OPD",
                     str_detect(patientVisit_DischargeOutcome, "Outpatient") ~ "OPD",
                     str_detect(patientVisit_DischargeOutcome, "GP") ~ "GP",
                     str_detect(patientVisit_DischargeOutcome, "Ambulatory ") ~ "Ambulatory",
                     str_detect(patientVisit_DischargeOutcome, "DIED") ~ "Died in Dept",
                     TRUE ~ "Other")) %>%
    mutate(adjusted_score = if_else(score %in% c(1, 2), 1.5, score),
```

```
          adjusted_scoref = as.factor(adjusted_score),
        across(c(dest, patient_GenderCode), as.factor),
        age = (as.numeric(difftime(document_CreatedWhen, patient_DOB, units = "days")))/365.25,
          time = ymd_hms(document_CreatedWhen),
          female = patient_GenderCode == "Female") %>%
left_join(fi_samples, by = c("patient_TrustNumber", "time")) %>%
  filter(!is.na(score),
        str_detect(patient_GenderCode , "ale"),
        !is.na(patient_TrustNumber),
        age >= age_filter,
        patientVisit_AttendanceNumber %in% unique_visitsage ) %>%
    left_join(news_per_visit)


#And those without scores
nocfsprocessed <- everyone_afterday1_age %>%
mutate(patientAddress_PostalCode = str_replace_all(patientAddress_PostalCode, " ", "") %>% str_to_upper()) %>%
  left_join(postcode_ref_2, by = "patientAddress_PostalCode") %>%
filter(    str_detect(patient_GenderCode , "ale"),
        !is.na(patient_TrustNumber) ) %>%
  mutate(female = patient_GenderCode == "Female",

    dest = case_when(str_detect(patientVisit_DischargeOutcome, "Admit") ~ "Admitted", #Summarise destinations
                    str_detect(patientVisit_DischargeOutcome, "Discharge") ~ "Home",
                    str_detect(patientVisit_DischargeOutcome, "Home|Left") ~ "Home",
                    str_detect(patientVisit_DischargeOutcome, "OPD|Clinic") ~ "OPD",
                    str_detect(patientVisit_DischargeOutcome, "Outpatient") ~ "OPD",
                    str_detect(patientVisit_DischargeOutcome, "GP") ~ "GP",
                    str_detect(patientVisit_DischargeOutcome, "Ambulatory ") ~ "Ambulatory",
                    str_detect(patientVisit_DischargeOutcome, "DIED") ~ "Died in Dept",
                    TRUE ~ "Other")) %>%
    mutate(across(c(dest, patient_GenderCode), as.factor),
        age = (as.numeric(difftime(document_CreatedWhen, patient_DOB, units = "days")))/365.25,) %>%
    left_join(news_per_visitnocfs) %>%
     filter( !patientVisit_AttendanceNumber %in% scores$patientVisit_AttendanceNumber)






#saved as combined_scores.csv
library(forcats)

# Add a column to distinguish between the two datasets
scores <- scores %>% mutate(Group = "With CFS")
```

```
scoresnocfs <- nocfsprocessed %>% mutate(Group = "No CFS")

# Combine the two datasets
combined_scores <- bind_rows(scores, scoresnocfs)
```

Tableone and table two

In [203…

```
# Define the function to map nationality codes to ONS ethnicity categories
map_ethnicity_function <- function(nationality_code) {
  ons_ethnicity <- case_when(
    nationality_code %in% c('White British', 'White Irish', 'White Scottish', 'White English', 'White Welsh', 'White Cornish',
                            'White and Black Caribben', 'White and Asian', 'White and Black African', 'White Nothern Irish',
                            'Other White Unspecified', 'Any other white back ground', 'Polish', 'Portuguese', 'Greek',
                            'Greek Cypriot', 'Cypriot (Part not stated)', 'Turkish Cypriot', 'Kosovan', 'All former USSR rep',
                            'Serbian', 'Croatian', 'Bosnian', 'Spanish', 'Italian', 'Albanian' ) ~ 'White',
    nationality_code %in% c('Black Caribbean', 'Black African', 'Black British', 'Any other Black background', 'Ghanaian',
                            'Nigerian', 'Other Black Unspecified', 'Black and Asian', 'Black and Chinese', 'Mixed Black',
                            'Mixed Asian', 'Black and White', 'Sudanese', 'Eritrean', 'Angolan', 'Somali') ~ 'Black',
    nationality_code %in% c('Asian/Chinese', 'Indian/British Indian', 'Pakistani/British Pakistani', 'Bangladeshi/British Bangladeshi',
                            'Other Asian Unspecified', 'Any other Asian background', 'East African Asian', 'Sri Lankan',
                            'Vietnamese', 'Punjabi', 'Sinhalese', 'Tamil', 'Chinese', 'Malaysian', 'Filipino', 'Japanese') ~ 'Asian',
    nationality_code %in% c('Mixed Black', 'Mixed Asian', 'Other Mixed', 'White and Black African', 'White and Black Caribbean',
                            'Chinese and White', 'Black and White') ~ 'Mixed',
    nationality_code %in% c('Arab', 'Middle East', 'Kurdish', 'Iranian', 'Iraqi', 'Ethiopian', 'Traveller', 'Gypsy/Romany',
                            'Other Latin American', 'Colombian', 'Ecuadorian') ~ 'Other',
    TRUE ~ 'Not Stated' # Catch-all for unspecified or unclear categories
  )

  return(ons_ethnicity)
}
los_data_1 <-  demo_data2 %>% filter(!is.na(clientvisit_dischargedtm))  %>% mutate(los = as.numeric(difftime(clientvisit_dischargedtm, clie

# Map ethnicity to ONS codes if not done yet
combined_data <- combined_scores %>%
  mutate(ons_ethnicity = map_ethnicity_function(patient_NationalityCode))%>%
group_by(Group) %>%
mutate(Unique_patients = n_distinct(patient_TrustNumber),
      Alive = is.na(patient_DeceasedDtm),
       clientvisit_visitidcode = gsub("[[:punct:]]", "", patientVisit_AttendanceNumber)) %>%
left_join(los_data_1) %>%
filter(!(PRUH == TRUE & document_CreatedWhen < ymd_hms("2018-02-12 11:26:00")))
```

```r
factorvars <- c('Group','female', 'presenting_complaint', 'ons_ethnicity', 'dest', 'PRUH', 'Alive')
continuous_vars <- c('age', 'NEWS_Score', 'adjusted_score', 'imd_rank', 'imd_decile', 'los', 'Unique_patients', 'filab')
tab1vars <- c(factorvars, continuous_vars)

table1 <- CreateTableOne(data = combined_data,
               strata = "Group",
               factorVars = factorvars, includeNA = TRUE,test = TRUE,addOverall = TRUE,
              vars = tab1vars,)

table1pruh <- table1 <- CreateTableOne(data = combined_data %>% filter(PRUH) %>% mutate(Unique_patients = n_distinct(patient_TrustNumber)),
               strata = "Group",
               factorVars = factorvars, includeNA = TRUE,test = TRUE,addOverall = TRUE,
              vars = tab1vars,)

table1dh <- table1 <- CreateTableOne(data = combined_data %>% filter(!PRUH) %>% mutate(Unique_patients = n_distinct(patient_TrustNumber)),
               strata = "Group",
               factorVars = factorvars, includeNA = TRUE,test = TRUE,addOverall = TRUE,
              vars = tab1vars,)

table1_df <- as.data.frame(print(table1, printToggle = TRUE))




write.csv(table1_df, "tableone_output.csv", row.names = TRUE)

# Calculate censorship time and create event time
combined_scores_unique <- combined_data %>%
  group_by(patient_TrustNumber) %>%
  mutate(
    censorship_time = max(document_CreatedWhen, na.rm = TRUE),
    event_time = ifelse(!is.na(patient_DeceasedDtm),
                        as.numeric(difftime(patient_DeceasedDtm, document_CreatedWhen, units = "days")),
                        as.numeric(difftime(censorship_time, document_CreatedWhen, units = "days")))
  ) %>%
  arrange(document_CreatedWhen) %>%
  slice(1) %>%
  ungroup() %>%
mutate(
    event = ifelse(!is.na(patient_DeceasedDtm), 1, 0)
  )
```

```
scores$age_scaled <- scale(scores$age)
scores$news_scaled <- scale(scores$NEWS_Score)
scores$filab_scaled <- scale(scores$filab)
scores$imd_scaled <- scale(scores$imd_rank)
scores$adjusted_score_scaled <- scale(scores$adjusted_score)

table2_data <- scores %>%
mutate(ons_ethnicity = map_ethnicity_function(patient_NationalityCode))%>%
group_by(adjusted_scoref) %>%
mutate(Unique_patients = n_distinct(patient_TrustNumber),
      Alive = is.na(patient_DeceasedDtm),
       clientvisit_visitidcode = gsub("[[:punct:]]", "", patientVisit_AttendanceNumber),
      nonews = is.na(NEWS_Score),
       nofilab = is.na(filab))%>%
left_join(los_data_1) %>%
mutate( year_month = floor_date(time, "month")) %>%
left_join(filabs)

factorvars <- c('Group','female', 'ons_ethnicity', 'dest', 'PRUH', 'Alive', 'nonews','nofilab')
continuous_vars <- c('age', 'NEWS_Score', 'imd_rank', 'imd_decile', 'los', 'Unique_patients', 'filab','fi')
tab1vars <- c(factorvars, continuous_vars)

table2 <- CreateTableOne(data = table2_data,
               strata = "adjusted_scoref",
               factorVars = factorvars, includeNA = TRUE,test = TRUE,addOverall = TRUE,
              vars = tab1vars)

table2_df <- as.data.frame(print(table2, printToggle = TRUE, missing = TRUE))




# save to CSV
write.csv(table2_df, "tableone_output2.csv", row.names = TRUE)
```

Post ED destinations

In [158…

```
#Group Denstinations
dest_data <- scores %>%
    select(patient_TrustNumber, patientVisit_AdmitReasonCode, patientVisit_RegistrationComplaint, patientVisit_DischargeOutcome, adjusted_s
    group_by(adjusted_score, dest) %>%
    summarise(n = n()) %>% arrange(adjusted_score, desc(n)) %>%
    group_by(dest) %>%
    mutate(total = sum(n)) %>%
    ungroup() %>%
```

```r
    mutate(destn = fct_reorder(dest, total, .desc = TRUE)) %>%
    group_by(adjusted_score) %>%
    mutate(rel_freq = n / sum(n)) %>%
    ungroup()



# Create a long format dataset for faceting
dest_data_long <- dest_data %>%
  pivot_longer(cols = c(n, rel_freq),
               names_to = "measure",
               values_to = "value") %>%
  mutate(measure = factor(measure, levels = c("n", "rel_freq"),
                          labels = c("Absolute Count", "Relative Frequency")))
options(repr.plot.width = 20, repr.plot.height = 10)
# Create the plot
ggplot(dest_data_long, aes(x = factor(adjusted_score), y = value, fill = dest)) +
  geom_bar(stat = "identity", position = "dodge") +
  scale_fill_brewer(palette = "Set3") +
  labs(x = "Adjusted Score", y = "Value", fill = "Destination") +
  theme_minimal() +
  theme(axis.text.x = element_text(angle = 45, hjust = 1)) +
  coord_flip() +
  facet_wrap(~ measure, scales = "free_x", ncol = 2) +
  scale_y_continuous(labels = scales::comma_format(accuracy = 0.01))
```

```
`summarise()` has grouped output by 'adjusted_score'. You can override using the `.groups` argument.
```

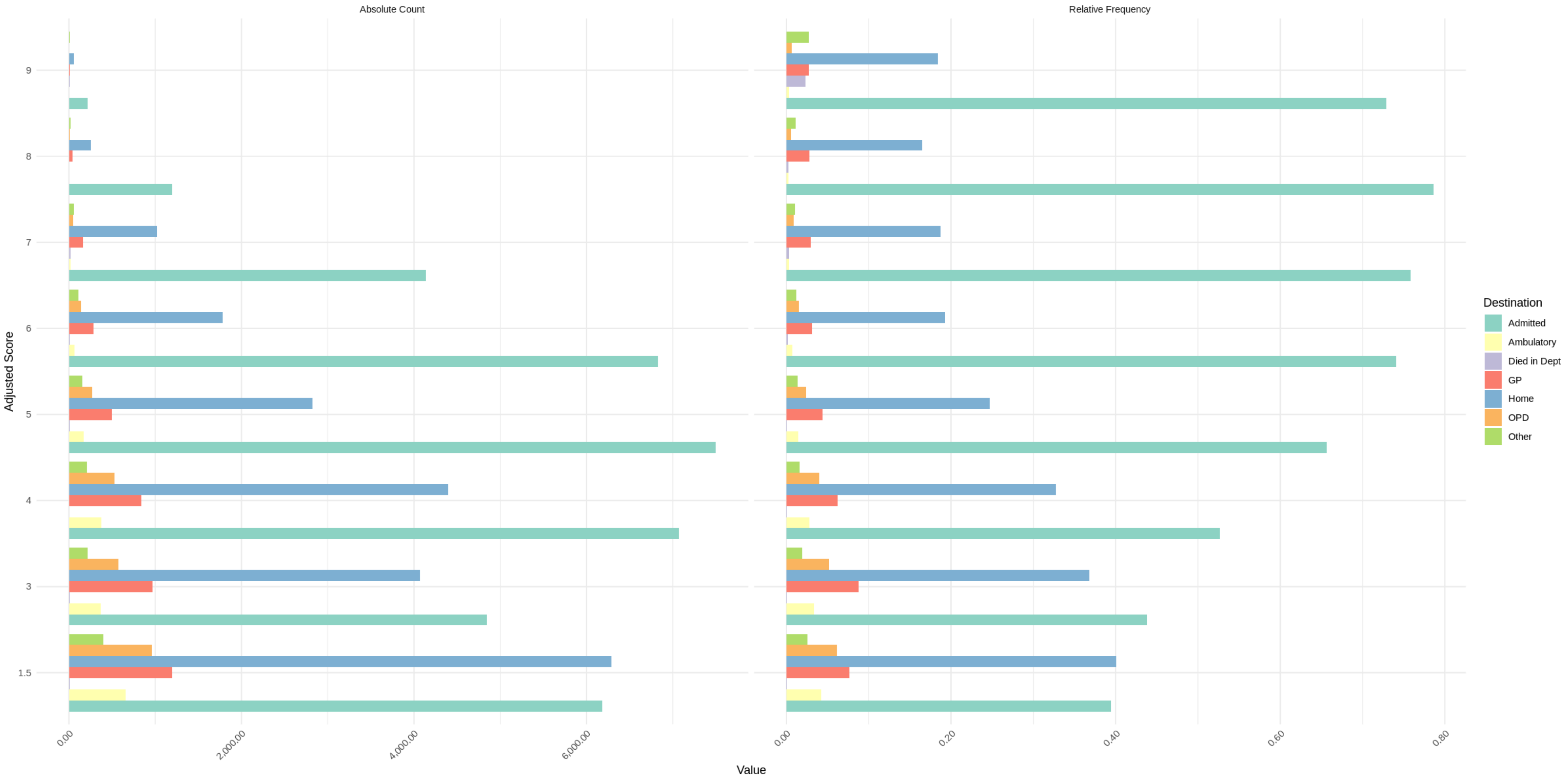

In [205…

```
# Fit the multinomial logistic regression model using GLM
multi_nom_model <- multinom(dest ~ adjusted_score_scaled + female + age_scaled + PRUH, data = scores)

multi_nom_model1 <- multinom(dest ~ adjusted_score_scaled + female + age_scaled + news_scaled + filab_scaled + imd_scaled + PRUH, data = sc


# Summary of the model
summary(multi_nom_model1)

# Tidy output with odds ratios and confidence intervals
tidy_results <- tidy(multi_nom_model1, conf.int = TRUE, exponentiate = TRUE)
print(tidy_results, n = Inf)
```

```
# weights:  42 (30 variable)
initial  value 132452.266116
iter  10 value 85256.269620
iter  20 value 84254.064376
iter  30 value 76900.444306
iter  40 value 72585.018503
iter  50 value 72547.590427
iter  60 value 72546.842672
iter  70 value 72546.624907
iter  80 value 72546.556105
iter  90 value 72546.227994
iter 100 value 72545.946572
final  value 72545.946572
stopped after 100 iterations
# weights:  63 (48 variable)
initial  value 55526.546103
iter  10 value 30983.561732
iter  20 value 30756.082446
iter  30 value 30048.495362
iter  40 value 29897.433449
iter  50 value 29812.529347
iter  60 value 29764.674783
iter  70 value 29762.924841
final  value 29762.923278
converged
```

```
Call:
multinom(formula = dest ~ adjusted_score_scaled + female + age_scaled + 
    news_scaled + filab_scaled + imd_scaled + PRUH, data = scores)

Coefficients:
             (Intercept) adjusted_score_scaled  femaleTRUE  age_scaled news_scaled filab_scaled  imd_scaled     PRUHTRUE
Ambulatory    -3.0810635            -0.8250719 -0.09211852 -0.31233084  -0.3032184   -0.1935094  0.04522366   0.16081108
Died in Dept -10.4036899             0.5597205 -1.67874175  0.07061357   0.5207719    0.6095800 -2.04876972   2.47615991
GP            -1.9853208            -0.6323648  0.21482655 -0.27296151  -0.3838052   -0.2967558 -0.06223558 -13.93563088
Home          -0.7125447            -0.4163635  0.08962749 -0.21343381  -0.3238490   -0.2781780  0.02018462   0.07534622
OPD           -2.8592057            -0.7521565 -0.04463207 -0.18283749  -0.4687550   -0.2923595  0.02364802  -0.17267313
Other         -4.0482653            -0.3766496 -0.16138488 -0.19782312  -0.2354196   -0.3158821  0.10373462   1.05871421

Std. Errors:
             (Intercept) adjusted_score_scaled femaleTRUE age_scaled news_scaled filab_scaled imd_scaled    PRUHTRUE
Ambulatory    0.06403895            0.04465698 0.07326663 0.04233851  0.04194165   0.03913212 0.04560935  0.10089894
Died in Dept  1.29356939            0.47296329 1.11354417 0.43779764  0.24109168   0.42363546 0.96637240  0.89345518
GP            0.04261774            0.02856876 0.05025442 0.02836876  0.02932636   0.02876270 0.03471902 41.01726611
Home          0.02429167            0.01604393 0.02868123 0.01574384  0.01556053   0.01556750 0.01823718  0.04167843
OPD           0.05887871            0.04090153 0.06866845 0.03879845  0.04401148   0.03782296 0.04340967  0.09907916
Other         0.09644244            0.06073926 0.10679276 0.05913457  0.05898373   0.05604610 0.06377636  0.13941237

Residual Deviance: 59525.85 
AIC: 59621.85
```

A tibble: 48 × 8

	y.level	term	estimate	std.error	statistic	p.value	conf.low	conf.high
	<chr>	<chr>	<dbl>	<dbl>	<dbl>	<dbl>	<dbl>	<dbl>
1	Ambulatory	(Intercept)	0.0459	0.0640	-48.1	0	4.05e- 2	5.21e- 2
2	Ambulatory	adjusted_score_scaled	0.438	0.0447	-18.5	3.24e- 76	4.01e- 1	4.78e- 1
3	Ambulatory	femaleTRUE	0.912	0.0733	-1.26	2.09e- 1	7.90e- 1	1.05e+ 0
4	Ambulatory	age_scaled	0.732	0.0423	-7.38	1.62e- 13	6.73e- 1	7.95e- 1
5	Ambulatory	news_scaled	0.738	0.0419	-7.23	4.85e- 13	6.80e- 1	8.02e- 1
6	Ambulatory	filab_scaled	0.824	0.0391	-4.95	7.61e- 7	7.63e- 1	8.90e- 1
7	Ambulatory	imd_scaled	1.05	0.0456	0.992	3.21e- 1	9.57e- 1	1.14e+ 0
8	Ambulatory	PRUHTRUE	1.17	0.101	1.59	1.11e- 1	9.64e- 1	1.43e+ 0
9	Died in Dept	(Intercept)	0.0000303	1.29	-8.04	8.79e- 16	2.40e- 6	3.83e- 4
10	Died in Dept	adjusted_score_scaled	1.75	0.473	1.18	2.37e- 1	6.93e- 1	4.42e+ 0
11	Died in Dept	femaleTRUE	0.187	1.11	-1.51	1.32e- 1	2.10e- 2	1.65e+ 0
12	Died in Dept	age_scaled	1.07	0.438	0.161	8.72e- 1	4.55e- 1	2.53e+ 0
13	Died in Dept	news_scaled	1.68	0.241	2.16	3.08e- 2	1.05e+ 0	2.70e+ 0
14	Died in Dept	filab_scaled	1.84	0.424	1.44	1.50e- 1	8.02e- 1	4.22e+ 0
15	Died in Dept	imd_scaled	0.129	0.966	-2.12	3.40e- 2	1.94e- 2	8.57e- 1
16	Died in Dept	PRUHTRUE	11.9	0.893	2.77	5.58e- 3	2.06e+ 0	6.85e+ 1
17	GP	(Intercept)	0.137	0.0426	-46.6	0	1.26e- 1	1.49e- 1
18	GP	adjusted_score_scaled	0.531	0.0286	-22.1	1.46e-108	5.02e- 1	5.62e- 1
19	GP	femaleTRUE	1.24	0.0503	4.27	1.91e- 5	1.12e+ 0	1.37e+ 0
20	GP	age_scaled	0.761	0.0284	-9.62	6.46e- 22	7.20e- 1	8.05e- 1
21	GP	news_scaled	0.681	0.0293	-13.1	3.89e- 39	6.43e- 1	7.22e- 1
22	GP	filab_scaled	0.743	0.0288	-10.3	5.88e- 25	7.02e- 1	7.86e- 1
23	GP	imd_scaled	0.940	0.0347	-1.79	7.30e- 2	8.78e- 1	1.01e+ 0
24	GP	PRUHTRUE	0.000000887	41.0	-0.340	7.34e- 1	1.08e-41	7.27e+28
25	Home	(Intercept)	0.490	0.0243	-29.3	3.95e-189	4.68e- 1	5.14e- 1
26	Home	adjusted_score_scaled	0.659	0.0160	-26.0	1.75e-148	6.39e- 1	6.81e- 1
27	Home	femaleTRUE	1.09	0.0287	3.12	1.78e- 3	1.03e+ 0	1.16e+ 0
28	Home	age_scaled	0.808	0.0157	-13.6	7.24e- 42	7.83e- 1	8.33e- 1
29	Home	news_scaled	0.723	0.0156	-20.8	3.36e- 96	7.02e- 1	7.46e- 1
30	Home	filab_scaled	0.757	0.0156	-17.9	2.05e- 71	7.34e- 1	7.81e- 1
31	Home	imd_scaled	1.02	0.0182	1.11	2.68e- 1	9.85e- 1	1.06e+ 0
32	Home	PRUHTRUE	1.08	0.0417	1.81	7.06e- 2	9.94e- 1	1.17e+ 0
33	OPD	(Intercept)	0.0573	0.0589	-48.6	0	5.11e- 2	6.43e- 2
34	OPD	adjusted_score_scaled	0.471	0.0409	-18.4	1.60e- 75	4.35e- 1	5.11e- 1
35	OPD	femaleTRUE	0.956	0.0687	-0.650	5.16e- 1	8.36e- 1	1.09e+ 0
36	OPD	age_scaled	0.833	0.0388	-4.71	2.45e- 6	7.72e- 1	8.99e- 1
37	OPD	news_scaled	0.626	0.0440	-10.7	1.73e- 26	5.74e- 1	6.82e- 1
38	OPD	filab_scaled	0.747	0.0378	-7.73	1.08e- 14	6.93e- 1	8.04e- 1
39	OPD	imd_scaled	1.02	0.0434	0.545	5.86e- 1	9.40e- 1	1.11e+ 0
40	OPD	PRUHTRUE	0.841	0.0991	-1.74	8.14e- 2	6.93e- 1	1.02e+ 0
41	Other	(Intercept)	0.0175	0.0964	-42.0	0	1.44e- 2	2.11e- 2
42	Other	adjusted_score_scaled	0.686	0.0607	-6.20	5.61e- 10	6.09e- 1	7.73e- 1
43	Other	femaleTRUE	0.851	0.107	-1.51	1.31e- 1	6.90e- 1	1.05e+ 0

```
44 Other       age_scaled            0.821         0.0591     -3.35  8.22e-  4 7.31e- 1  9.21e- 1
45 Other       news_scaled           0.790         0.0590     -3.99  6.57e-  5 7.04e- 1  8.87e- 1
46 Other       filab_scaled          0.729         0.0560     -5.64  1.74e-  8 6.53e- 1  8.14e- 1
47 Other       imd_scaled            1.11          0.0638      1.63  1.04e-  1 9.79e- 1  1.26e+ 0
48 Other       PRUHTRUE              2.88          0.139       7.59  3.10e- 14 2.19e+ 0  3.79e+ 0
```

In [207…

```
# Summary of the model
summary(multi_nom_model)

# Tidy output with odds ratios and confidence intervals
tidy_results <- tidy(multi_nom_model, conf.int = TRUE, exponentiate = TRUE)
print(tidy_results, n = Inf)
```

```
Call:
multinom(formula = dest ~ adjusted_score_scaled + female + age_scaled +
    PRUH, data = scores)

Coefficients:
             (Intercept) adjusted_score_scaled  femaleTRUE age_scaled     PRUHTRUE
Ambulatory    -3.3072265            -0.8328812  0.01897122 -0.3319315  -0.05809630
Died in Dept  -6.2858807             0.4950826 -0.15645588  0.1003244  -0.43018261
GP            -1.9319413            -0.7196902  0.22931348 -0.2166850 -14.77347919
Home          -0.6749297            -0.4918136  0.10163645 -0.1791659   0.01261657
OPD           -2.8490966            -0.7806059  0.15728022 -0.2808097  -0.22629334
Other         -3.8176706            -0.4348527 -0.09696345 -0.1822569   0.79519728

Std. Errors:
             (Intercept) adjusted_score_scaled femaleTRUE  age_scaled    PRUHTRUE
Ambulatory    0.04541506           0.030732287 0.05094696 0.028629541  0.05341703
Died in Dept  0.20511659           0.130202646 0.23297188 0.117793265  0.27675028
GP            0.02736729           0.019574001 0.03483956 0.019101707 30.05700803
Home          0.01514266           0.009767535 0.01798241 0.009440249  0.01886452
OPD           0.03687968           0.024623096 0.04201751 0.023115357  0.04458308
Other         0.05582830           0.033102766 0.05996479 0.031883652  0.06261656

Residual Deviance: 145091.9
AIC: 145151.9
```

A tibble: 30 × 8

	y.level	term	estimate	std.error	statistic	p.value	conf.low	conf.high
	<chr>	<chr>	<dbl>	<dbl>	<dbl>	<dbl>	<dbl>	<dbl>
1	Ambulatory	(Intercept)	0.0366	0.0454	-72.8	0	3.35e- 2	4.00e- 2
2	Ambulatory	adjusted_score_scaled	0.435	0.0307	-27.1	9.54e-162	4.09e- 1	4.62e- 1
3	Ambulatory	femaleTRUE	1.02	0.0509	0.372	7.10e- 1	9.22e- 1	1.13e+ 0
4	Ambulatory	age_scaled	0.718	0.0286	-11.6	4.42e- 31	6.78e- 1	7.59e- 1
5	Ambulatory	PRUHTRUE	0.944	0.0534	-1.09	2.77e- 1	8.50e- 1	1.05e+ 0
6	Died in Dept	(Intercept)	0.00186	0.205	-30.6	3.04e-206	1.25e- 3	2.78e- 3
7	Died in Dept	adjusted_score_scaled	1.64	0.130	3.80	1.43e- 4	1.27e+ 0	2.12e+ 0
8	Died in Dept	femaleTRUE	0.855	0.233	-0.672	5.02e- 1	5.42e- 1	1.35e+ 0
9	Died in Dept	age_scaled	1.11	0.118	0.852	3.94e- 1	8.78e- 1	1.39e+ 0
10	Died in Dept	PRUHTRUE	0.650	0.277	-1.55	1.20e- 1	3.78e- 1	1.12e+ 0
11	GP	(Intercept)	0.145	0.0274	-70.6	0	1.37e- 1	1.53e- 1
12	GP	adjusted_score_scaled	0.487	0.0196	-36.8	6.07e-296	4.69e- 1	5.06e- 1
13	GP	femaleTRUE	1.26	0.0348	6.58	4.64e- 11	1.17e+ 0	1.35e+ 0
14	GP	age_scaled	0.805	0.0191	-11.3	7.97e- 30	7.76e- 1	8.36e- 1
15	GP	PRUHTRUE	0.000000384	30.1	-0.492	6.23e- 1	9.99e-33	1.47e+19
16	Home	(Intercept)	0.509	0.0151	-44.6	0	4.94e- 1	5.25e- 1
17	Home	adjusted_score_scaled	0.612	0.00977	-50.4	0	6.00e- 1	6.23e- 1
18	Home	femaleTRUE	1.11	0.0180	5.65	1.59e- 8	1.07e+ 0	1.15e+ 0
19	Home	age_scaled	0.836	0.00944	-19.0	2.55e- 80	8.21e- 1	8.52e- 1
20	Home	PRUHTRUE	1.01	0.0189	0.669	5.04e- 1	9.76e- 1	1.05e+ 0
21	OPD	(Intercept)	0.0579	0.0369	-77.3	0	5.39e- 2	6.22e- 2
22	OPD	adjusted_score_scaled	0.458	0.0246	-31.7	1.45e-220	4.37e- 1	4.81e- 1
23	OPD	femaleTRUE	1.17	0.0420	3.74	1.82e- 4	1.08e+ 0	1.27e+ 0
24	OPD	age_scaled	0.755	0.0231	-12.1	5.87e- 34	7.22e- 1	7.90e- 1
25	OPD	PRUHTRUE	0.797	0.0446	-5.08	3.86e- 7	7.31e- 1	8.70e- 1
26	Other	(Intercept)	0.0220	0.0558	-68.4	0	1.97e- 2	2.45e- 2
27	Other	adjusted_score_scaled	0.647	0.0331	-13.1	2.04e- 39	6.07e- 1	6.91e- 1
28	Other	femaleTRUE	0.908	0.0600	-1.62	1.06e- 1	8.07e- 1	1.02e+ 0
29	Other	age_scaled	0.833	0.0319	-5.72	1.09e- 8	7.83e- 1	8.87e- 1
30	Other	PRUHTRUE	2.21	0.0626	12.7	5.95e- 37	1.96e+ 0	2.50e+ 0

Cox setup

In [22]:

```
discharge_times <- demo_data2 %>% filter(clientvisit_visitidcode %in% table2_data$clientvisit_visitidcode) %>%
group_by(client_idcode) %>%
mutate(updatetime = max(updatetime)) %>%
group_by(client_idcode, clientvisit_visitidcode ) %>%
summarise(clientvisit_dischargedtm = first(clientvisit_dischargedtm), dod = first(client_deceaseddtm), updatetime = max(updatetime) )

los_surv <- table2_data  %>%
    #rename(client_idcode = patient_TrustNumber) %>%
    left_join(discharge_times, by = c('client_idcode', 'clientvisit_visitidcode')) %>%
```

```
    arrange(time) %>%
    group_by(client_idcode) %>%
    mutate(last_update = max(max(time, na.rm = TRUE), max(updatetime, na.rm = TRUE)),
           dod = coalesce(dod, last_update),
    died_ip = patient_DeceasedDtm < (clientvisit_dischargedtm + hours(24)),
          died_ip = if_else(is.na(died_ip), FALSE,died_ip))

los_surv$fiscaled <- scale(los_surv$fi)

los_surv$figroups <- cut(los_surv$fi, breaks = 8)
```

Cox LOS

In [20]:

```
#Unscaled
#Complete Cases
cox_model_mega_los <- coxph(Surv(los, !died_ip) ~ adjusted_score + NEWS_Score + filab + age + female + PRUH + imd_decile , data = los_surv)
#Full Dataset
cox_model_los <- coxph(Surv(los, !died_ip) ~ adjusted_score + age + female + PRUH, data = los_surv)
#Scaled
cox_model_mega_los_scaled <- coxph(Surv(los, !died_ip) ~ adjusted_score_scaled + news_scaled + filab_scaled + age_scaled + female + PRUH + [illegible]
cox_model_los_scaled <- coxph(Surv(los, !died_ip) ~ adjusted_score_scaled + age_scaled + female + PRUH, data = los_surv)

summary(cox_model_mega_los)
summary(cox_model_los)
summary(cox_model_mega_los_scaled)
summary(cox_model_los_scaled)
```

```
Call:
coxph(formula = Surv(los, !died_ip) ~ adjusted_score + NEWS_Score +
    filab + age + female + PRUH + imd_decile, data = los_surv)

  n= 13860, number of events= 13671
   (17442 observations deleted due to missingness)

                    coef exp(coef)  se(coef)       z Pr(>|z|)
adjusted_score -0.116329  0.890183  0.004985 -23.336   <2e-16 ***
NEWS_Score     -0.076902  0.925980  0.006296 -12.214   <2e-16 ***
filab          -1.236298  0.290458  0.082818 -14.928   <2e-16 ***
age            -0.011053  0.989008  0.001282  -8.623   <2e-16 ***
femaleTRUE      0.016349  1.016483  0.017301   0.945    0.345
PRUHTRUE        0.041822  1.042709  0.026036   1.606    0.108
imd_decile     -0.003911  0.996096  0.004188  -0.934    0.350
---
Signif. codes:  0 ‘***’ 0.001 ‘**’ 0.01 ‘*’ 0.05 ‘.’ 0.1 ‘ ’ 1

               exp(coef) exp(-coef) lower .95 upper .95
adjusted_score    0.8902     1.1234    0.8815    0.8989
NEWS_Score        0.9260     1.0799    0.9146    0.9375
filab             0.2905     3.4428    0.2469    0.3416
age               0.9890     1.0111    0.9865    0.9915
femaleTRUE        1.0165     0.9838    0.9826    1.0515
PRUHTRUE          1.0427     0.9590    0.9908    1.0973
imd_decile        0.9961     1.0039    0.9880    1.0043

Concordance= 0.613  (se = 0.003 )
Likelihood ratio test= 1297  on 7 df,   p=<2e-16
Wald test            = 1292  on 7 df,   p=<2e-16
Score (logrank) test = 1299  on 7 df,   p=<2e-16
```

```
Call:
coxph(formula = Surv(los, !died_ip) ~ adjusted_score + age +
    female + PRUH, data = los_surv)

  n= 30187, number of events= 29654
   (1115 observations deleted due to missingness)

                     coef  exp(coef)   se(coef)       z Pr(>|z|)
adjusted_score -0.1324391  0.8759563  0.0032838 -40.331   <2e-16 ***
age            -0.0099805  0.9900692  0.0008502 -11.739   <2e-16 ***
femaleTRUE      0.0184102  1.0185808  0.0117609   1.565    0.117
PRUHTRUE       -0.0001716  0.9998285  0.0134097  -0.013    0.990
---
Signif. codes:  0 ‘***’ 0.001 ‘**’ 0.01 ‘*’ 0.05 ‘.’ 0.1 ‘ ’ 1

               exp(coef) exp(-coef) lower .95 upper .95
adjusted_score    0.8760     1.1416    0.8703    0.8816
age               0.9901     1.0100    0.9884    0.9917
femaleTRUE        1.0186     0.9818    0.9954    1.0423
PRUHTRUE          0.9998     1.0002    0.9739    1.0265

Concordance= 0.6  (se = 0.002 )
Likelihood ratio test= 2194  on 4 df,   p=<2e-16
Wald test            = 2185  on 4 df,   p=<2e-16
Score (logrank) test = 2207  on 4 df,   p=<2e-16
```

```
Call:
coxph(formula = Surv(los, !died_ip) ~ adjusted_score_scaled +
    news_scaled + filab_scaled + age_scaled + female + PRUH +
    imd_scaled, data = los_surv)

  n= 13860, number of events= 13671
   (17442 observations deleted due to missingness)

                          coef exp(coef)  se(coef)       z Pr(>|z|)
adjusted_score_scaled -0.217585  0.804459  0.009323 -23.339   <2e-16 ***
news_scaled           -0.100991  0.903941  0.008270 -12.212   <2e-16 ***
filab_scaled          -0.137342  0.871672  0.009202 -14.926   <2e-16 ***
age_scaled            -0.080069  0.923052  0.009286  -8.622   <2e-16 ***
femaleTRUE             0.016287  1.016420  0.017301   0.941    0.347
PRUHTRUE               0.042200  1.043103  0.026130   1.615    0.106
imd_scaled            -0.010218  0.989834  0.010766  -0.949    0.343
---
Signif. codes:  0 ‘***’ 0.001 ‘**’ 0.01 ‘*’ 0.05 ‘.’ 0.1 ‘ ’ 1

                      exp(coef) exp(-coef) lower .95 upper .95
adjusted_score_scaled    0.8045     1.2431    0.7899    0.8193
news_scaled              0.9039     1.1063    0.8894    0.9187
filab_scaled             0.8717     1.1472    0.8561    0.8875
age_scaled               0.9231     1.0834    0.9064    0.9400
femaleTRUE               1.0164     0.9838    0.9825    1.0515
PRUHTRUE                 1.0431     0.9587    0.9910    1.0979
imd_scaled               0.9898     1.0103    0.9692    1.0109

Concordance= 0.613  (se = 0.003 )
Likelihood ratio test= 1297  on 7 df,   p=<2e-16
Wald test            = 1293  on 7 df,   p=<2e-16
Score (logrank) test = 1299  on 7 df,   p=<2e-16
```

```
Call:
coxph(formula = Surv(los, !died_ip) ~ adjusted_score_scaled + 
    age_scaled + female + PRUH, data = los_surv)

  n= 30187, number of events= 29654 
   (1115 observations deleted due to missingness)

                            coef  exp(coef)   se(coef)       z Pr(>|z|)    
adjusted_score_scaled -0.2477152  0.7805822  0.0061420 -40.331   <2e-16 ***
age_scaled            -0.0723088  0.9302436  0.0061599 -11.739   <2e-16 ***
femaleTRUE             0.0184102  1.0185808  0.0117609   1.565    0.117    
PRUHTRUE              -0.0001716  0.9998285  0.0134097  -0.013    0.990    
---
Signif. codes:  0 ‘***’ 0.001 ‘**’ 0.01 ‘*’ 0.05 ‘.’ 0.1 ‘ ’ 1

                      exp(coef) exp(-coef) lower .95 upper .95
adjusted_score_scaled    0.7806     1.2811    0.7712    0.7900
age_scaled               0.9302     1.0750    0.9191    0.9415
femaleTRUE               1.0186     0.9818    0.9954    1.0423
PRUHTRUE                 0.9998     1.0002    0.9739    1.0265

Concordance= 0.6  (se = 0.002 )
Likelihood ratio test= 2194  on 4 df,   p=<2e-16
Wald test            = 2185  on 4 df,   p=<2e-16
Score (logrank) test = 2207  on 4 df,   p=<2e-16
```

In [306…

```
los_fit_cfs <- survfit(Surv(los, !died_ip) ~ adjusted_scoref, data = los_surv)
# Create a named vector of colors
custom_palette <- setNames(brewer.pal(8, "Set1"), levels(los_surv$adjusted_scoref))

# Plot Kaplan-Meier curve with modifications
km_plot <- ggsurvplot(
  los_fit_cfs,
  data = los_surv,
  pval = FALSE,
  risk.table = FALSE,
  fontsize = 4,
  ggtheme = theme_minimal(),
  palette = custom_palette,
  legend.title = "Frailty Score",
   legend.labs = levels(los_surv$adjusted_scoref),
  xlab = "Days",
  ylab = "Discharge Probability",
  conf.int = FALSE,
  xlim = c(0, 10),
  ylim = c(0, 1),
```

```r
  break.time.by = 1,
  legend = "right",
  font.family = "sans",
  font.legend = 10,
  font.x = 12,
  font.y = 12,
  font.tickslab = 10,
  linetype = "solid",
  censor = FALSE,
fun = "event"
)

# Further customization
km_plot$plot <- km_plot$plot +
  theme(
    legend.position = c(0.85, 0.75),
    legend.background = element_rect(fill = "white", color = "black"),
    panel.grid.major = element_blank(),
    panel.grid.minor = element_blank(),
    axis.line = element_line(color = "black"),
    plot.title = element_text(hjust = 0.5, size = 14, face = "bold"),
    axis.title = element_text(size = 12, face = "bold"),
    axis.text = element_text(size = 10)
  )

# Display the plot with optimal width and height for Jupyter Notebook
options(repr.plot.width = 12, repr.plot.height = 8)
print(km_plot)
```

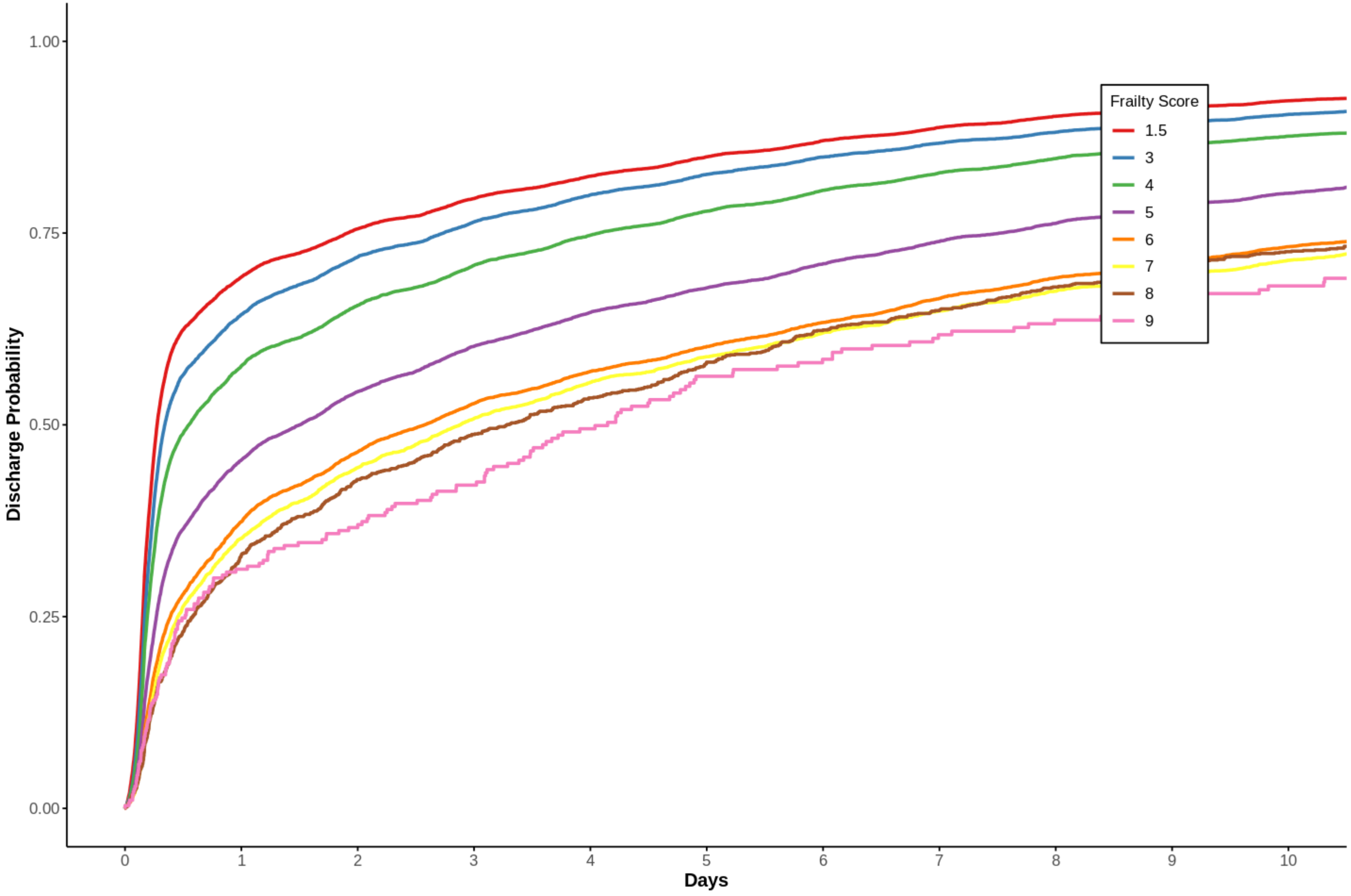

Cox IP deaths

In [21]:
```
IP_deaths <- los_surv %>% group_by(client_idcode) %>% arrange(desc(time)) %>% slice(1L) %>% mutate(died_ip = if_else(los > 30, FALSE, died_
                                                                                                                       los = min(los, 30))
```

In [22]:
```
cox_model_IP_cfs <- coxph(Surv(los, died_ip) ~ adjusted_score  + age + female + PRUH, data = IP_deaths)
cox_model_IP_cfs_scaled <- coxph(Surv(los, died_ip) ~ adjusted_score_scaled  + age_scaled + female + PRUH, data = IP_deaths)
cox_model_mega_IP_scaled <- coxph(Surv(los, died_ip) ~ adjusted_score_scaled + news_scaled + filab_scaled + age_scaled + female + PRUH + im
cox_model_mega_IP <- coxph(Surv(los, died_ip) ~ adjusted_score + NEWS_Score + filab + age + female + PRUH + imd_decile , data = IP_deaths)



summary(cox_model_IP_cfs)
summary(cox_model_IP_cfs_scaled)
```

```r
summary(cox_model_mega_IP)
summary(cox_model_mega_IP_scaled)
```

```
Call:
coxph(formula = Surv(los, died_ip) ~ adjusted_score + age + female + 
    PRUH, data = IP_deaths)

  n= 10137, number of events= 428 
   (469 observations deleted due to missingness)

                    coef exp(coef)  se(coef)      z Pr(>|z|)    
adjusted_score  0.180313  1.197592  0.030229  5.965 2.45e-09 ***
age             0.011031  1.011092  0.006845  1.612   0.1071    
femaleTRUE     -0.307265  0.735455  0.097362 -3.156   0.0016 ** 
PRUHTRUE       -0.494205  0.610055  0.125806 -3.928 8.55e-05 ***
---
Signif. codes:  0 ‘***’ 0.001 ‘**’ 0.01 ‘*’ 0.05 ‘.’ 0.1 ‘ ’ 1

               exp(coef) exp(-coef) lower .95 upper .95
adjusted_score    1.1976      0.835    1.1287    1.2707
age               1.0111      0.989    0.9976    1.0247
femaleTRUE        0.7355      1.360    0.6077    0.8901
PRUHTRUE          0.6101      1.639    0.4767    0.7806

Concordance= 0.669  (se = 0.016 )
Likelihood ratio test= 90.45  on 4 df,   p=<2e-16
Wald test            = 82.63  on 4 df,   p=<2e-16
Score (logrank) test = 84.56  on 4 df,   p=<2e-16
```

```
Call:
coxph(formula = Surv(los, died_ip) ~ adjusted_score_scaled + 
    age_scaled + female + PRUH, data = IP_deaths)

  n= 10137, number of events= 428 
   (469 observations deleted due to missingness)

                          coef exp(coef) se(coef)      z Pr(>|z|)    
adjusted_score_scaled  0.33726   1.40110  0.05654  5.965 2.45e-09 ***
age_scaled             0.07992   1.08320  0.04959  1.612   0.1071    
femaleTRUE            -0.30727   0.73546  0.09736 -3.156   0.0016 ** 
PRUHTRUE              -0.49421   0.61006  0.12581 -3.928 8.55e-05 ***
---
Signif. codes:  0 '***' 0.001 '**' 0.01 '*' 0.05 '.' 0.1 ' ' 1

                      exp(coef) exp(-coef) lower .95 upper .95
adjusted_score_scaled    1.4011     0.7137    1.2541    1.5653
age_scaled               1.0832     0.9232    0.9829    1.1938
femaleTRUE               0.7355     1.3597    0.6077    0.8901
PRUHTRUE                 0.6101     1.6392    0.4767    0.7806

Concordance= 0.669  (se = 0.016 )
Likelihood ratio test= 90.45  on 4 df,   p=<2e-16
Wald test            = 82.63  on 4 df,   p=<2e-16
Score (logrank) test = 84.56  on 4 df,   p=<2e-16
```

```
Call:
coxph(formula = Surv(los, died_ip) ~ adjusted_score + NEWS_Score + 
    filab + age + female + PRUH + imd_decile, data = IP_deaths)

  n= 4318, number of events= 136 
   (6288 observations deleted due to missingness)

                    coef  exp(coef)   se(coef)      z Pr(>|z|)    
adjusted_score   0.142541   1.153201   0.055227  2.581  0.00985 ** 
NEWS_Score       0.184091   1.202126   0.045684  4.030 5.59e-05 ***
filab            4.761428 116.912808   0.804195  5.921 3.21e-09 ***
age              0.018867   1.019046   0.012684  1.487  0.13689    
femaleTRUE      -0.565231   0.568229   0.175675 -3.217  0.00129 ** 
PRUHTRUE        -0.586300   0.556382   0.344907 -1.700  0.08915 .  
imd_decile       0.001391   1.001392   0.043458  0.032  0.97446    
---
Signif. codes:  0 ‘***’ 0.001 ‘**’ 0.01 ‘*’ 0.05 ‘.’ 0.1 ‘ ’ 1

               exp(coef) exp(-coef) lower .95 upper .95
adjusted_score    1.1532   0.867152    1.0349    1.2850
NEWS_Score        1.2021   0.831860    1.0992    1.3147
filab           116.9128   0.008553   24.1730  565.4502
age               1.0190   0.981310    0.9940    1.0447
femaleTRUE        0.5682   1.759855    0.4027    0.8018
PRUHTRUE          0.5564   1.797326    0.2830    1.0939
imd_decile        1.0014   0.998610    0.9196    1.0904

Concordance= 0.748  (se = 0.022 )
Likelihood ratio test= 81.3  on 7 df,   p=7e-15
Wald test            = 76.23  on 7 df,   p=8e-14
Score (logrank) test = 79.26  on 7 df,   p=2e-14
```

```
Call:
coxph(formula = Surv(los, died_ip) ~ adjusted_score_scaled +
    news_scaled + filab_scaled + age_scaled + female + PRUH +
    imd_scaled, data = IP_deaths)

  n= 4318, number of events= 136
   (6288 observations deleted due to missingness)

                           coef exp(coef)  se(coef)      z Pr(>|z|)
adjusted_score_scaled  0.265654  1.304284  0.103197  2.574  0.01005 *
news_scaled            0.241534  1.273200  0.059991  4.026 5.67e-05 ***
filab_scaled           0.529376  1.697872  0.089371  5.923 3.15e-09 ***
age_scaled             0.137305  1.147178  0.091914  1.494  0.13521
femaleTRUE            -0.565501  0.568075  0.175693 -3.219  0.00129 **
PRUHTRUE              -0.573928  0.563308  0.346121 -1.658  0.09728 .
imd_scaled            -0.006968  0.993056  0.112509 -0.062  0.95061
---
Signif. codes:  0 ‘***’ 0.001 ‘**’ 0.01 ‘*’ 0.05 ‘.’ 0.1 ‘ ’ 1

                      exp(coef) exp(-coef) lower .95 upper .95
adjusted_score_scaled    1.3043     0.7667    1.0654    1.5967
news_scaled              1.2732     0.7854    1.1320    1.4321
filab_scaled             1.6979     0.5890    1.4251    2.0229
age_scaled               1.1472     0.8717    0.9581    1.3736
femaleTRUE               0.5681     1.7603    0.4026    0.8016
PRUHTRUE                 0.5633     1.7752    0.2858    1.1101
imd_scaled               0.9931     1.0070    0.7965    1.2381

Concordance= 0.747  (se = 0.022 )
Likelihood ratio test= 81.3  on 7 df,   p=7e-15
Wald test            = 76.23  on 7 df,   p=8e-14
Score (logrank) test = 79.26  on 7 df,   p=2e-14
```

In [26]:

```
fit_cfs_IP <- survfit(Surv(los, died_ip) ~ adjusted_score , data = IP_deaths)

# Create a named vector of colors
custom_palette <- setNames(brewer.pal(8, "Set1"), levels(IP_deaths$adjusted_score))

# Plot Kaplan-Meier curve with modifications
km_plot <- ggsurvplot(
  fit_cfs_IP,
  data = IP_deaths,
  pval = FALSE,
  risk.table = FALSE,
  fontsize = 4,
  ggtheme = theme_minimal(),
```

```r
  palette = custom_palette,
  legend.title = "Frailty Score",
   legend.labs = levels(IP_deaths$adjusted_score),
  xlab = "Days Elapsed",
  ylab = "Survival Probability",
  conf.int = FALSE,
  xlim = c(0, 30),
  ylim = c(0, 1),
  break.time.by = 5,
  legend = "right",
  font.family = "sans",
  font.legend = 10,
  font.x = 12,
  font.y = 12,
  font.tickslab = 10,
  linetype = "solid",
  censor = FALSE
)

# Further customization
km_plot$plot <- km_plot$plot +
  theme(
    legend.position = c(0.85, 0.75),
    legend.background = element_rect(fill = "white", color = "black"),
    panel.grid.major = element_blank(),
    panel.grid.minor = element_blank(),
    axis.line = element_line(color = "black"),
    plot.title = element_text(hjust = 0.5, size = 14, face = "bold"),
    axis.title = element_text(size = 12, face = "bold"),
    axis.text = element_text(size = 10)
  )

# Display the plot with optimal width and height for Jupyter Notebook
options(repr.plot.width = 12, repr.plot.height = 8)
print(km_plot)
```

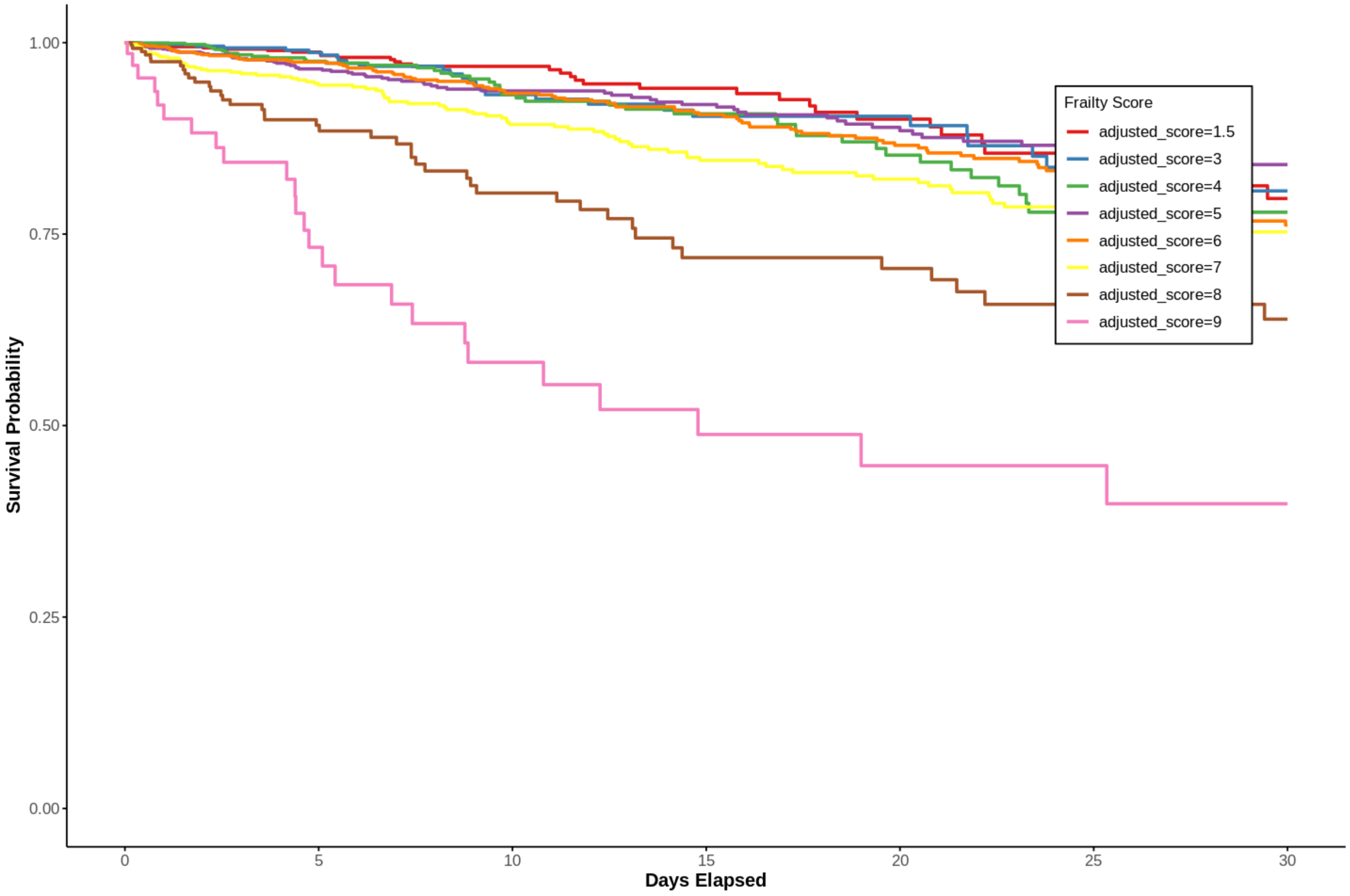

Readmission Models

In [30]:

```
#Create readmit cohort
readmit_surv <- los_surv %>%
group_by(patient_TrustNumber) %>%
arrange(document_CreatedWhen) %>%
mutate(time_to_revisit = as.numeric(difftime(lead(document_CreatedWhen), document_CreatedWhen, units = "days")),
       revisit_date = lead(document_CreatedWhen),
      time_to_end = as.numeric(difftime(last_update, document_CreatedWhen, units = "days")),
      readmitted = !is.na(time_to_revisit),
      time_to_revisit2 = if_else(is.na(time_to_revisit), time_to_end, time_to_revisit)) %>%
mutate(PRUH = str_detect(patientVisit_AttendanceNumber, "PR"))

cc_cohort <- readmit_surv %>%
group_by(patient_TrustNumber) %>%
arrange(document_CreatedWhen) %>%
```

```r
  slice(1) %>%
  ungroup() %>%
mutate(
    status = case_when(
     !is.na(revisit_date) ~ 1,
      Alive ~ 2,
      TRUE ~ 0
    ),
    follow_up_time = case_when(
      status == 1 ~ time_to_revisit,
      status == 2 ~ time_to_end,
      TRUE ~ time_to_end
    )
  )
```

In [33]:
```r
simple_fg <- crr(
  ftime = cc_cohort$follow_up_time,
  fstatus = cc_cohort$status,
  cov1 =cc_cohort[c('adjusted_score', 'age', 'PRUH', 'female' )],
  cencode = 1
)
summary(simple_fg)
```

```
Competing Risks Regression

Call:
crr(ftime = cc_cohort$follow_up_time, fstatus = cc_cohort$status,
    cov1 = cc_cohort[c("adjusted_score", "age", "PRUH", "female")],
    cencode = 1)

                  coef exp(coef) se(coef)     z p-value
adjusted_score  0.0443     1.045  0.00668  6.64 3.1e-11
age             0.0159     1.016  0.00171  9.29 0.0e+00
PRUH           -0.2268     0.797  0.02877 -7.88 3.1e-15
female         -0.0636     0.938  0.02503 -2.54 1.1e-02

               exp(coef) exp(-coef)  2.5% 97.5%
adjusted_score     1.045      0.957 1.032 1.059
age                1.016      0.984 1.013 1.019
PRUH               0.797      1.255 0.753 0.843
female             0.938      1.066 0.893 0.986

Num. cases = 17620
Pseudo Log-likelihood = -60931
Pseudo likelihood ratio test = 235  on 4 df,
```

In [31]:

```
complex_fg <- crr(
  ftime = cc_cohort$follow_up_time,
  fstatus = cc_cohort$status,
  cov1 =cc_cohort[c('adjusted_score', 'age', 'PRUH', 'female', 'NEWS_Score','filab', 'imd_decile' )],
  cencode = 1
)
summary(complex_fg)
```

```
11868 cases omitted due to missing values
Competing Risks Regression

Call:
crr(ftime = cc_cohort$follow_up_time, fstatus = cc_cohort$status,
    cov1 = cc_cohort[c("adjusted_score", "age", "PRUH", "female",
        "NEWS_Score", "filab", "imd_decile")], cencode = 1)

                  coef exp(coef) se(coef)      z p-value
adjusted_score  0.0423     1.043  0.00906  4.667 3.1e-06
age             0.0132     1.013  0.00235  5.610 2.0e-08
PRUH            0.0848     1.089  0.05009  1.694 9.0e-02
female         -0.0462     0.955  0.03174 -1.457 1.5e-01
NEWS_Score      0.0395     1.040  0.01140  3.462 5.4e-04
filab           0.6543     1.924  0.15195  4.306 1.7e-05
imd_decile      0.0046     1.005  0.00770  0.597 5.5e-01

               exp(coef) exp(-coef)  2.5% 97.5%
adjusted_score     1.043      0.959 1.025  1.06
age                1.013      0.987 1.009  1.02
PRUH               1.089      0.919 0.987  1.20
female             0.955      1.047 0.897  1.02
NEWS_Score         1.040      0.961 1.017  1.06
filab              1.924      0.520 1.428  2.59
imd_decile         1.005      0.995 0.990  1.02

Num. cases = 5752 (11868 cases omitted due to missing values)
Pseudo Log-likelihood = -32953
Pseudo likelihood ratio test = 111  on 7 df,
```

In [34]:

```
complex_fg_scaled <- crr(
  ftime = cc_cohort$follow_up_time,
  fstatus = cc_cohort$status,
  cov1 =cc_cohort[c('adjusted_score_scaled', 'age_scaled', 'PRUH', 'female', 'news_scaled', 'filab_scaled', 'imd_scaled' )],
  cencode = 1
)
summary(complex_fg_scaled)
```

```
11868 cases omitted due to missing values
Competing Risks Regression

Call:
crr(ftime = cc_cohort$follow_up_time, fstatus = cc_cohort$status,
    cov1 = cc_cohort[c("adjusted_score_scaled", "age_scaled",
        "PRUH", "female", "news_scaled", "filab_scaled", "imd_scaled")],
    cencode = 1)

                         coef exp(coef) se(coef)      z p-value
adjusted_score_scaled  0.0790     1.082   0.0169  4.663 3.1e-06
age_scaled             0.0957     1.100   0.0170  5.616 2.0e-08
PRUH                   0.0863     1.090   0.0502  1.719 8.6e-02
female                -0.0463     0.955   0.0317 -1.458 1.4e-01
news_scaled            0.0518     1.053   0.0150  3.461 5.4e-04
filab_scaled           0.0727     1.075   0.0169  4.306 1.7e-05
imd_scaled             0.0105     1.011   0.0198  0.531 6.0e-01

                      exp(coef) exp(-coef)  2.5% 97.5%
adjusted_score_scaled     1.082      0.924 1.047  1.12
age_scaled                1.100      0.909 1.064  1.14
PRUH                      1.090      0.917 0.988  1.20
female                    0.955      1.047 0.897  1.02
news_scaled               1.053      0.950 1.023  1.08
filab_scaled              1.075      0.930 1.040  1.11
imd_scaled                1.011      0.990 0.972  1.05

Num. cases = 5752 (11868 cases omitted due to missing values)
Pseudo Log-likelihood = -32953
Pseudo likelihood ratio test = 111  on 7 df,
```

Deaths outside Hospital

In [35]:

```
all_deaths <- los_surv %>% group_by(client_idcode) %>% arrange(time) %>% slice(1L)%>% mutate(days_elapsed = as.numeric(difftime(dod, time,
```

In [329…

```
cox_model_OP_cfs <- coxph(Surv(days_elapsed, !Alive) ~ adjusted_score  + age + female + PRUH, data = all_deaths)
cox_model_OP_cfs_scaled <- coxph(Surv(days_elapsed, !Alive) ~ adjusted_score_scaled  + age_scaled + female + PRUH, data = all_deaths)

cox_model_mega_OP_scaled <- coxph(Surv(days_elapsed, !Alive) ~ adjusted_score_scaled + news_scaled + filab_scaled + age_scaled + female + P
cox_model_mega_OP <- coxph(Surv(days_elapsed, !Alive) ~ adjusted_score + NEWS_Score + filab + age + female + PRUH + imd_decile , data = all



summary(cox_model_OP_cfs)
summary(cox_model_OP_cfs_scaled)
```

```r
summary(cox_model_mega_OP)
summary(cox_model_mega_OP_scaled)
```

```
Call:
coxph(formula = Surv(days_elapsed, !Alive) ~ adjusted_score +
    age + female + PRUH, data = all_deaths)

  n= 33475, number of events= 8871

                    coef exp(coef)  se(coef)      z Pr(>|z|)
adjusted_score  0.221577  1.248043  0.006089  36.39   <2e-16 ***
age             0.043836  1.044811  0.001522  28.79   <2e-16 ***
femaleTRUE     -0.347098  0.706736  0.021425 -16.20   <2e-16 ***
PRUHTRUE        0.284003  1.328436  0.022187  12.80   <2e-16 ***
---
Signif. codes:  0 '***' 0.001 '**' 0.01 '*' 0.05 '.' 0.1 ' ' 1

               exp(coef) exp(-coef) lower .95 upper .95
adjusted_score    1.2480     0.8013    1.2332     1.263
age               1.0448     0.9571    1.0417     1.048
femaleTRUE        0.7067     1.4150    0.6777     0.737
PRUHTRUE          1.3284     0.7528    1.2719     1.387

Concordance= 0.661  (se = 0.003 )
Likelihood ratio test= 3000  on 4 df,   p=<2e-16
Wald test            = 2981  on 4 df,   p=<2e-16
Score (logrank) test = 3061  on 4 df,   p=<2e-16
```

```
Call:
coxph(formula = Surv(days_elapsed, !Alive) ~ adjusted_score_scaled +
    age_scaled + female + PRUH, data = all_deaths)

  n= 33475, number of events= 8871

                          coef exp(coef) se(coef)      z Pr(>|z|)
adjusted_score_scaled  0.41444   1.51352  0.01139  36.39   <2e-16 ***
age_scaled             0.31759   1.37382  0.01103  28.79   <2e-16 ***
femaleTRUE            -0.34710   0.70674  0.02143 -16.20   <2e-16 ***
PRUHTRUE               0.28400   1.32844  0.02219  12.80   <2e-16 ***
---
Signif. codes:  0 ‘***’ 0.001 ‘**’ 0.01 ‘*’ 0.05 ‘.’ 0.1 ‘ ’ 1

                      exp(coef) exp(-coef) lower .95 upper .95
adjusted_score_scaled    1.5135     0.6607    1.4801     1.548
age_scaled               1.3738     0.7279    1.3444     1.404
femaleTRUE               0.7067     1.4150    0.6777     0.737
PRUHTRUE                 1.3284     0.7528    1.2719     1.387

Concordance= 0.661  (se = 0.003 )
Likelihood ratio test= 3000  on 4 df,   p=<2e-16
Wald test            = 2981  on 4 df,   p=<2e-16
Score (logrank) test = 3061  on 4 df,   p=<2e-16
```

```
Call:
coxph(formula = Surv(days_elapsed, !Alive) ~ adjusted_score +
    NEWS_Score + filab + age + female + PRUH + imd_decile, data = all_deaths)

  n= 8282, number of events= 2647
   (25193 observations deleted due to missingness)

                    coef exp(coef)  se(coef)      z Pr(>|z|)
adjusted_score  0.194323  1.214488  0.011607 16.742  < 2e-16 ***
NEWS_Score      0.101666  1.107014  0.013141  7.737 1.02e-14 ***
filab           1.360498  3.898133  0.181990  7.476 7.68e-14 ***
age             0.042886  1.043819  0.002899 14.791  < 2e-16 ***
femaleTRUE     -0.342252  0.710169  0.039250 -8.720  < 2e-16 ***
PRUHTRUE        0.453597  1.573964  0.054731  8.288  < 2e-16 ***
imd_decile     -0.010102  0.989949  0.009292 -1.087    0.277
---
Signif. codes:  0 ‘***’ 0.001 ‘**’ 0.01 ‘*’ 0.05 ‘.’ 0.1 ‘ ’ 1

               exp(coef) exp(-coef) lower .95 upper .95
adjusted_score    1.2145     0.8234    1.1872     1.242
NEWS_Score        1.1070     0.9033    1.0789     1.136
filab             3.8981     0.2565    2.7286     5.569
age               1.0438     0.9580    1.0379     1.050
femaleTRUE        0.7102     1.4081    0.6576     0.767
PRUHTRUE          1.5740     0.6353    1.4139     1.752
imd_decile        0.9899     1.0102    0.9721     1.008

Concordance= 0.663  (se = 0.006 )
Likelihood ratio test= 868  on 7 df,   p=<2e-16
Wald test            = 879.9  on 7 df,   p=<2e-16
Score (logrank) test = 887.3  on 7 df,   p=<2e-16
```

```
Call:
coxph(formula = Surv(days_elapsed, !Alive) ~ adjusted_score_scaled +
    news_scaled + filab_scaled + age_scaled + female + PRUH +
    imd_scaled, data = all_deaths)

  n= 8282, number of events= 2647
   (25193 observations deleted due to missingness)

                          coef exp(coef) se(coef)      z Pr(>|z|)
adjusted_score_scaled  0.36340   1.43821  0.02171 16.740  < 2e-16 ***
news_scaled            0.13348   1.14280  0.01726  7.734 1.04e-14 ***
filab_scaled           0.15123   1.16327  0.02022  7.479 7.49e-14 ***
age_scaled             0.31076   1.36447  0.02101 14.795  < 2e-16 ***
femaleTRUE            -0.34224   0.71018  0.03925 -8.720  < 2e-16 ***
PRUHTRUE               0.45635   1.57831  0.05484  8.321  < 2e-16 ***
imd_scaled            -0.02874   0.97167  0.02449 -1.173    0.241
---
Signif. codes:  0 '***' 0.001 '**' 0.01 '*' 0.05 '.' 0.1 ' ' 1

                      exp(coef) exp(-coef) lower .95 upper .95
adjusted_score_scaled    1.4382     0.6953    1.3783     1.501
news_scaled              1.1428     0.8750    1.1048     1.182
filab_scaled             1.1633     0.8596    1.1181     1.210
age_scaled               1.3645     0.7329    1.3094     1.422
femaleTRUE               0.7102     1.4081    0.6576     0.767
PRUHTRUE                 1.5783     0.6336    1.4175     1.757
imd_scaled               0.9717     1.0292    0.9261     1.019

Concordance= 0.663  (se = 0.006 )
Likelihood ratio test= 868.2  on 7 df,   p=<2e-16
Wald test            = 880.1  on 7 df,   p=<2e-16
Score (logrank) test = 887.5  on 7 df,   p=<2e-16
```

In [40]:
```
all_deaths1 <- all_deaths %>%
  mutate(days_censored = pmin(days_elapsed, 90),
         status_censored = ifelse(days_elapsed > 90, 0, !Alive))

fit_90 <- survfit(Surv(days_censored, status_censored) ~ adjusted_scoref, data = all_deaths1)
```

In [36]:
```
fit_cfs <- survfit(Surv(days_elapsed, !Alive) ~ adjusted_scoref, data = all_deaths)
```

In [41]:
```
# Create a named vector of colors
custom_palette <- setNames(brewer.pal(8, "Set1"), levels(all_deaths1$adjusted_scoref))

# Plot Kaplan-Meier curve with modifications
km_plot <- ggsurvplot(
```

```
  fit_90,
  data = all_deaths1,
  pval = FALSE,
  risk.table = FALSE,
  fontsize = 4,
  ggtheme = theme_minimal(),
  palette = custom_palette,
  legend.title = "Frailty Score",
   legend.labs = levels(all_deaths1$adjusted_scoref),
  xlab = "Days since ED Attendance",
  ylab = "Survival Probability",
  conf.int = FALSE,
  xlim = c(0, 90),
  ylim = c(0, 1),
  break.time.by = 5,
  legend = "right",
  font.family = "sans",
  font.legend = 10,
  font.x = 12,
  font.y = 12,
  font.tickslab = 10,
  linetype = "solid",
  censor = TRUE
)

# Further customization
km_plot$plot <- km_plot$plot +
  theme(
    legend.position = c(0.85, 0.75),
    legend.background = element_rect(fill = "white", color = "black"),
    panel.grid.major = element_blank(),
    panel.grid.minor = element_blank(),
    axis.line = element_line(color = "black"),
    plot.title = element_text(hjust = 0.5, size = 14, face = "bold"),
    axis.title = element_text(size = 12, face = "bold"),
    axis.text = element_text(size = 10)
  )

# Display the plot with optimal width and height for Jupyter Notebook
options(repr.plot.width = 12, repr.plot.height = 8)
print(km_plot)
```

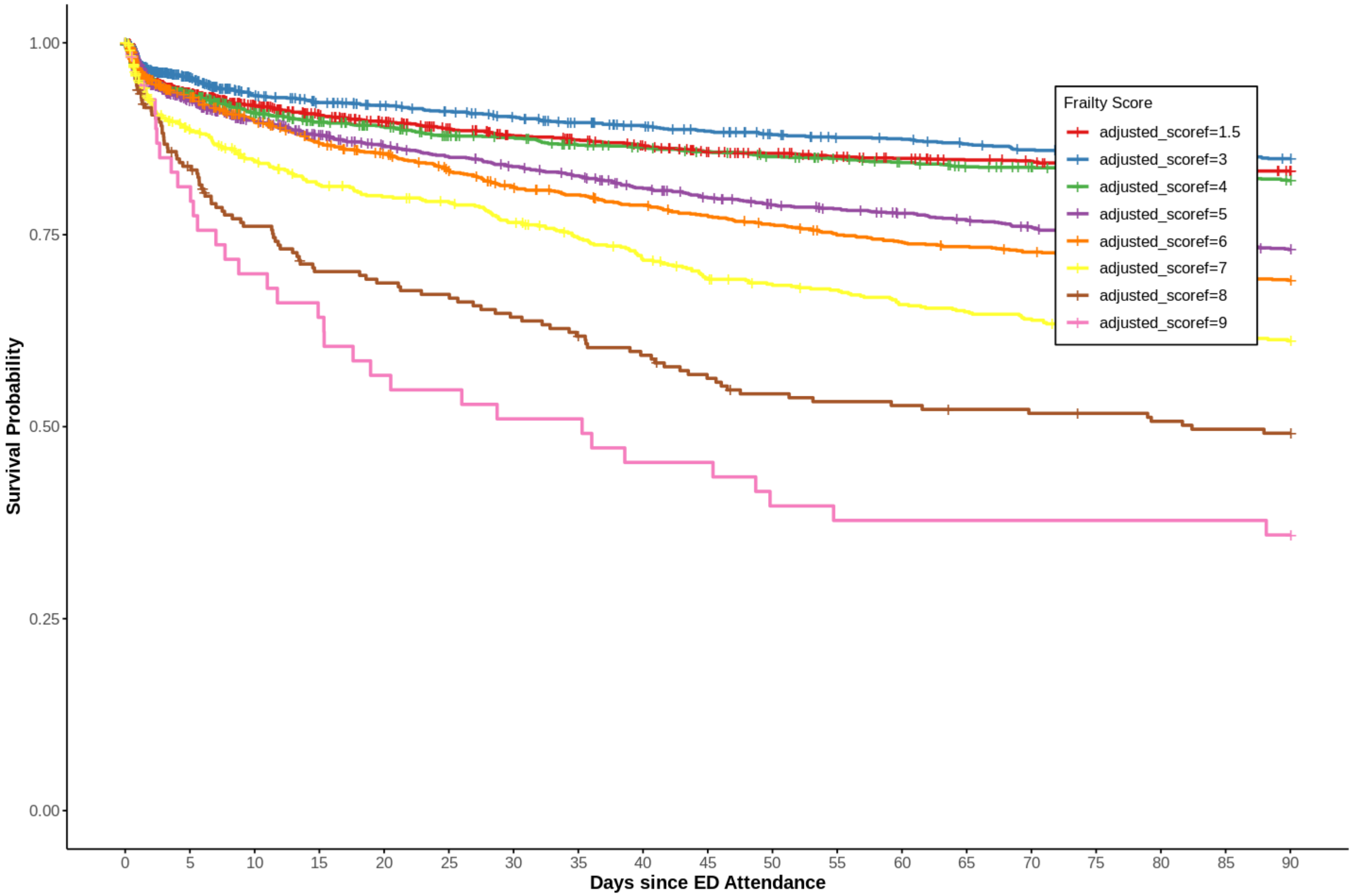

In [38]:

```
# Create a named vector of colors
custom_palette <- setNames(brewer.pal(8, "Set1"), levels(all_deaths$adjusted_scoref))

# Plot Kaplan-Meier curve with modifications
km_plot <- ggsurvplot(
  fit_cfs,
  data = all_deaths,
  pval = FALSE,
  risk.table = FALSE,
  fontsize = 4,
  ggtheme = theme_minimal(),
  palette = custom_palette,
  legend.title = "Frailty Score",
   legend.labs = levels(all_deaths$adjusted_scoref),
  xlab = "Days Elapsed",
  ylab = "Survival Probability",
  conf.int = FALSE,
```

```r
  xlim = c(0, 1000),
  ylim = c(0, 1),
  break.time.by = 200,
  legend = "right",
  font.family = "sans",
  font.legend = 10,
  font.x = 12,
  font.y = 12,
  font.tickslab = 10,
  linetype = "solid",
  censor = TRUE
)

# Further customization
km_plot$plot <- km_plot$plot +
  theme(
    legend.position = c(0.85, 0.75),
    legend.background = element_rect(fill = "white", color = "black"),
    panel.grid.major = element_blank(),
    panel.grid.minor = element_blank(),
    axis.line = element_line(color = "black"),
    plot.title = element_text(hjust = 0.5, size = 14, face = "bold"),
    axis.title = element_text(size = 12, face = "bold"),
    axis.text = element_text(size = 10)
  )

# Display the plot with optimal width and height for Jupyter Notebook
options(repr.plot.width = 12, repr.plot.height = 8)
print(km_plot)
```

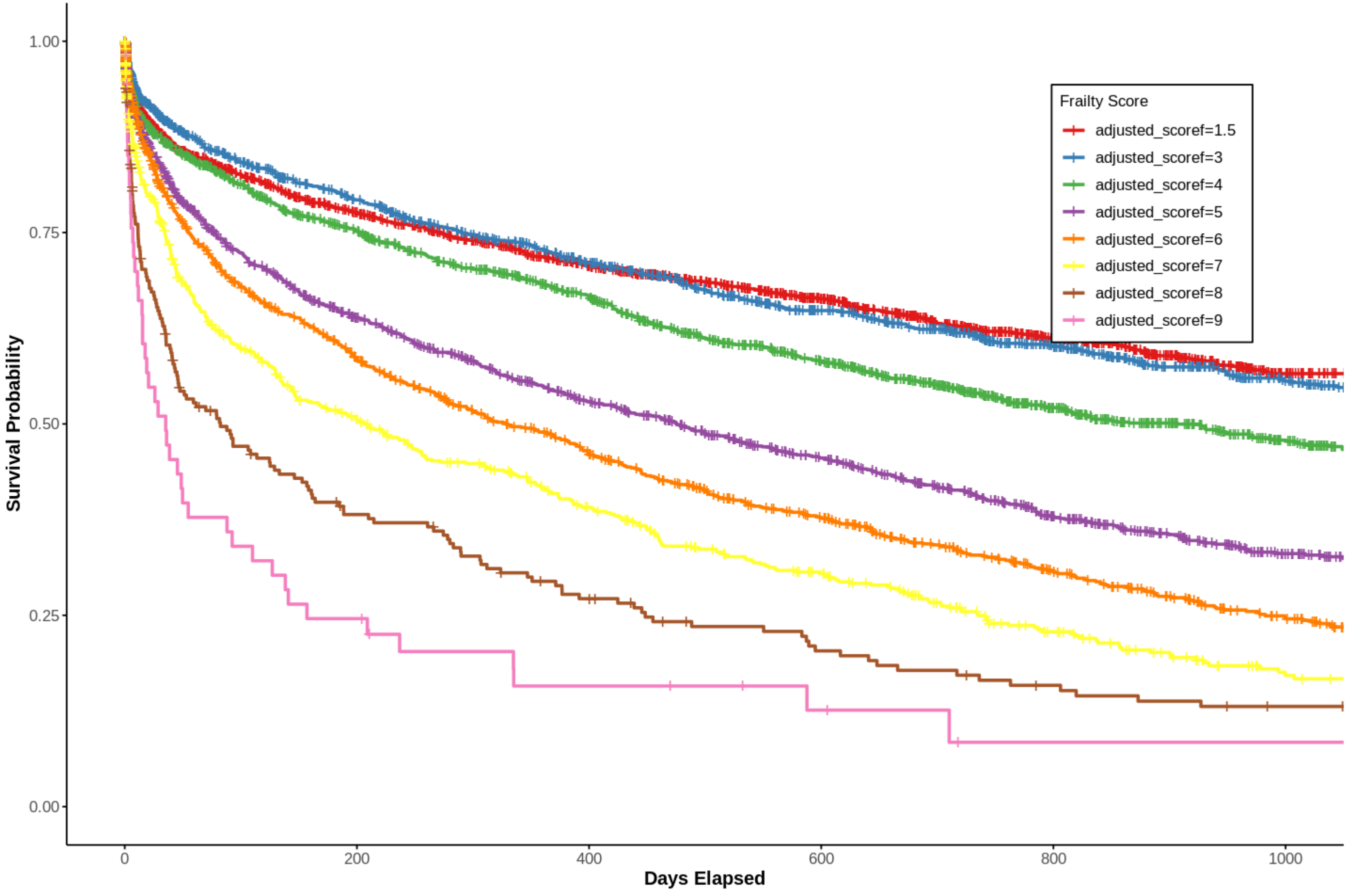

Multiple Measurement

In [ ]:

```
multi_measurements3 <- scores %>%
  group_by(patient_TrustNumber) %>%
  #filter(n() > 1) %>%
    arrange(document_CreatedWhen)  %>%
mutate(measurement = row_number(),
       start_time = min(document_CreatedWhen),
      time_passed = document_CreatedWhen - start_time,
       time = ymd_hms(document_CreatedWhen))
```

In [281…

```
multi_measurements3 <- table2_data %>%
 group_by(patient_TrustNumber) %>%
 # filter(n() > 1) %>%
    arrange(time)  %>%
mutate(measurement = row_number(),
```

```
      start_time = min(time),
     time_passed = time - start_time)
```

In [379…

```
lag_times <- multi_measurements3 %>%
select(patient_TrustNumber, document_CreatedWhen, adjusted_score, score) %>%
arrange(document_CreatedWhen) %>%
group_by(patient_TrustNumber) %>%
mutate(lag_time = as.numeric(difftime(document_CreatedWhen, lag(document_CreatedWhen),, units = "days")),
       score_delta = score - lag(score)) %>%
arrange(patient_TrustNumber, document_CreatedWhen)
```

In [387…

```
lag_times %>%
  ggplot(aes(x = lag_time, y = jitter(score_delta))) +
  geom_point(alpha = 0.04) +
  theme_bw() +
  labs(x = "Time between readings (days)",
       y = "Difference between scores") +
  scale_y_continuous(breaks = seq(floor(min(lag_times$score_delta, na.rm = TRUE)),
                                  ceiling(max(lag_times$score_delta, na.rm = TRUE)),
                                  by = 1))
```

```
Warning message:
“Removed 33488 rows containing missing values (`geom_point()`).”
```

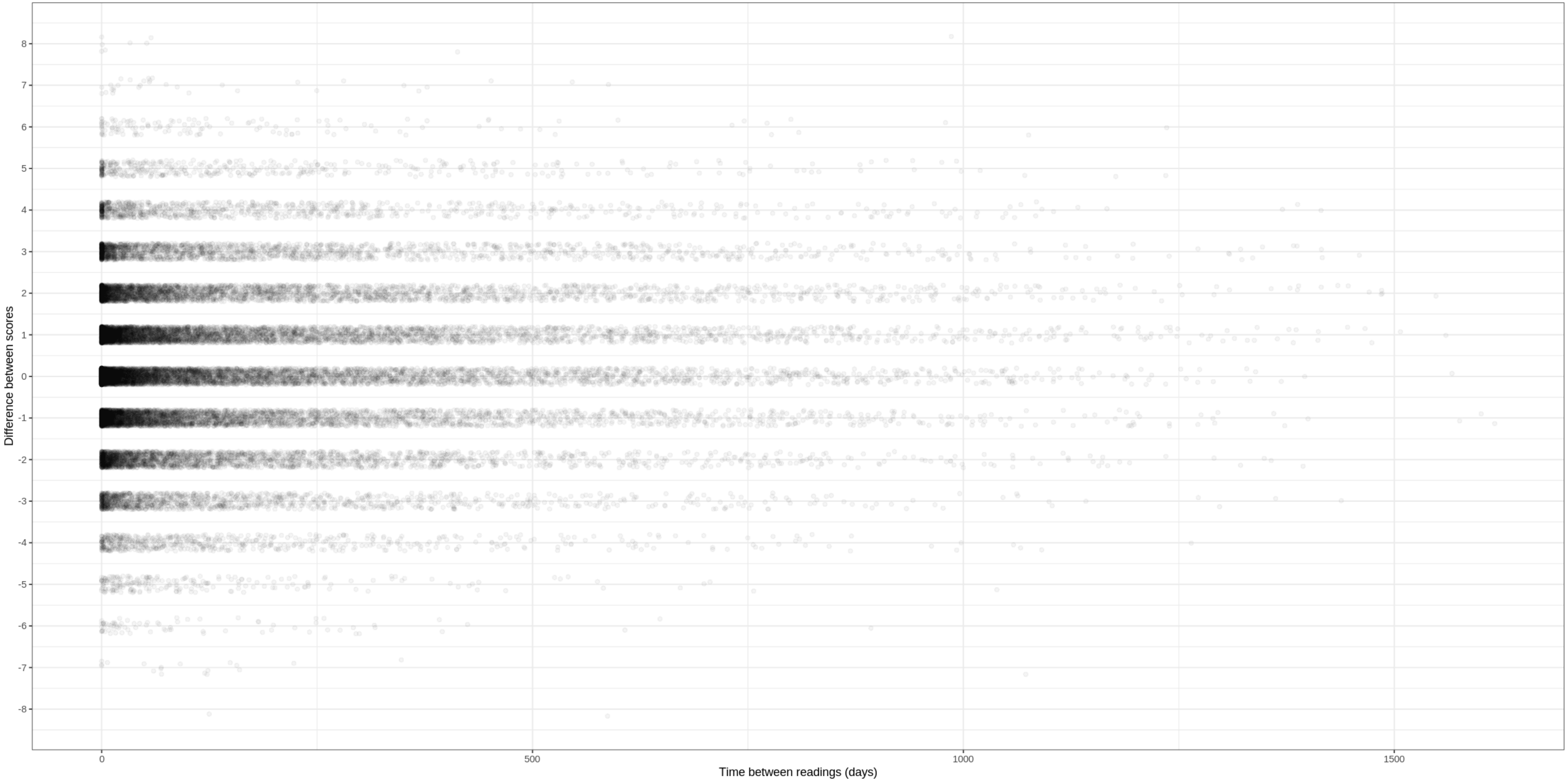

In [ ]:

```
graphdata <- multi_measurements3 %>%
left_join(chon_fi_labs) %>%
ungroup() %>%
  mutate( years_passed = as.numeric(time_passed)/(365.25*24*60*60),
    anonymous_id = as.factor(dense_rank(patient_TrustNumber)),
  ) %>%
group_by(anonymous_id)%>%
filter(max(measurement) > 30)
```

In [289…

```
custom_palette <- c(
  "#E41A1C", "#377EB8", "#4DAF4A", "#984EA3", "#FF7F00", "#1E90FF",
  "#A65628", "#F781BF", "#999999", "#66C2A5", "#FC8D62", "#8DA0CB"
)
options(repr.plot.width = 20, repr.plot.height = 10)
graphdata %>%
```

```
ggplot(aes(x = years_passed, y = score, colour = anonymous_id)) +
geom_point(size = 2, alpha = 0.9) +
  facet_wrap(~anonymous_id) +
scale_color_manual(values = custom_palette) +  # More distinct color palette
  scale_x_continuous(breaks = function(x) seq(floor(min(x)), ceiling(max(x)), by = 1)) +
  scale_y_continuous(limits = c(0, 9), breaks = seq(0, 9, by = 1)) +  # Set y-axis from 0 to 9
  labs(
    x = "Years since index admission",
    y = "CFS Score",
    title = "Patient Scores Over Time",
    subtitle = "Each panel represents an individual patient"
  ) +
                     theme_minimal() +
  theme(
    legend.position = "none",
    strip.background = element_blank(),
    strip.text = element_blank(),
    panel.border = element_rect(colour = "grey80", fill = NA),
    panel.grid.minor = element_blank(),
    axis.text = element_text(size = 10),
    axis.title = element_text(size = 12, face = "bold"),
    plot.title = element_text(size = 14, face = "bold", hjust = 0.5),
    plot.subtitle = element_text(size = 11, hjust = 0.5),
    plot.margin = unit(c(1, 1, 1, 1), "cm")
  )
```

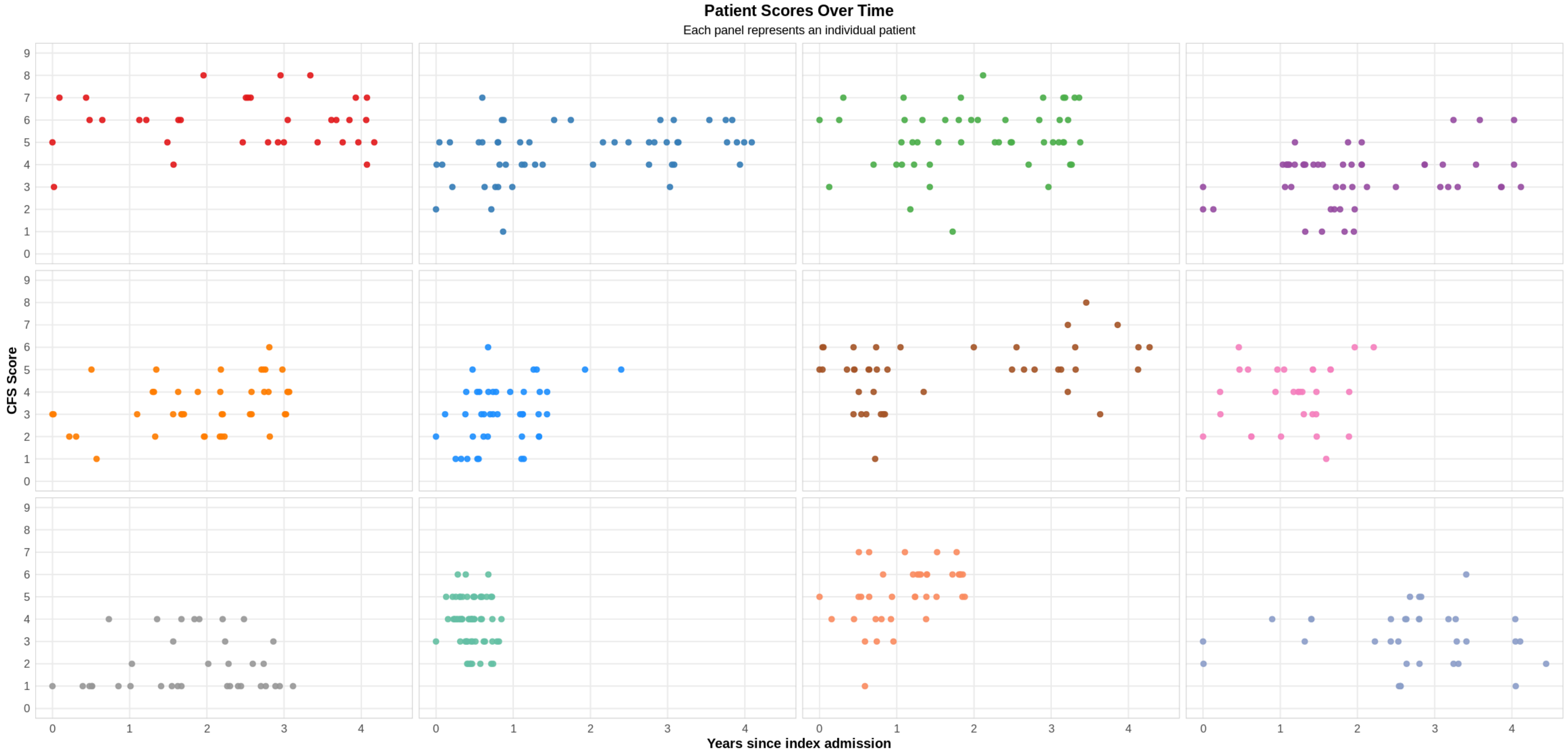

Random effects models

In [352…

```
smaller_multi$rater <- as.factor(smaller_multi$rater)

model_simple <- lmer(adjusted_score ~ female + age + PRUH + (1 | patient_TrustNumber) + (1  | rater), data = smaller_multi)
summary(model_simple)
```

```
Linear mixed model fit by REML ['lmerMod']
Formula: adjusted_score ~ female + age + PRUH + (1 | patient_TrustNumber) +      (1 | rater)
   Data: smaller_multi

REML criterion at convergence: 251576

Scaled residuals: 
    Min      1Q  Median      3Q     Max 
-3.7865 -0.5862 -0.0258  0.5589  5.6301 

Random effects:
 Groups              Name        Variance Std.Dev.
 patient_TrustNumber (Intercept) 1.0733   1.0360  
 rater               (Intercept) 0.6246   0.7903  
 Residual                        1.5878   1.2601  
Number of obs: 68067, groups:  patient_TrustNumber, 33488; rater, 491

Fixed effects:
             Estimate Std. Error t value
(Intercept) -1.873332   0.101591 -18.440
femaleTRUE   0.062923   0.016055   3.919
age          0.074702   0.001088  68.636
PRUHTRUE    -0.762188   0.097376  -7.827

Correlation of Fixed Effects:
           (Intr) fmTRUE age   
femaleTRUE -0.016              
age        -0.863 -0.082       
PRUHTRUE   -0.236 -0.001 -0.024
```

In [353…

```
model_pc <- lmer(adjusted_score ~ female + age + PRUH + presenting_complaint + (1 | patient_TrustNumber) + (1  | rater), data = smaller_mul
summary(model_pc)
```

```
Correlation matrix not shown by default, as p = 36 > 12.
Use print(obj, correlation=TRUE)  or
    vcov(obj)        if you need it
```

```
Linear mixed model fit by REML ['lmerMod']
Formula: adjusted_score ~ female + age + PRUH + presenting_complaint +      (1 | patient_TrustNumber) + (1 | rater)
   Data: smaller_multi

REML criterion at convergence: 250132

Scaled residuals: 
    Min      1Q  Median      3Q     Max 
-3.5727 -0.5908 -0.0229  0.5640  5.7172 

Random effects:
 Groups              Name        Variance Std.Dev.
 patient_TrustNumber (Intercept) 0.9765   0.9882  
 rater               (Intercept) 0.6116   0.7820  
 Residual                        1.5886   1.2604  
Number of obs: 68067, groups:  patient_TrustNumber, 33488; rater, 491

Fixed effects:
                                                                      Estimate Std. Error t value
(Intercept)                                                          -1.208124   0.102833 -11.748
femaleTRUE                                                            0.078430   0.015803   4.963
age                                                                   0.070338   0.001073  65.568
PRUHTRUE                                                             -0.796736   0.096393  -8.266
presenting_complaintAirway / breathing                               -0.188241   0.117035  -1.608
presenting_complaintAirway / breathing >> Difficulty breathing        0.109040   0.048882   2.231
presenting_complaintAirway / breathing >> Short of breath            -0.226382   0.028948  -7.820
presenting_complaintCirculation / chest                              -0.362633   0.055087  -6.583
presenting_complaintCirculation / chest >> Chest pain                -0.696544   0.030454 -22.872
presenting_complaintCirculation / chest >> Collapse / fainting episode   -0.457357   0.049181  -9.299
presenting_complaintCirculation / chest >> Palpitations              -0.738858   0.053795 -13.735
presenting_complaintEnvironmental                                    -0.592840   0.135777  -4.366
presenting_complaintEye                                              -0.742960   0.075493  -9.841
presenting_complaintGastrointestinal                                 -0.193195   0.033350  -5.793
presenting_complaintGastrointestinal >> Abdominal pain               -0.461012   0.031537 -14.618
presenting_complaintGeneral / minor / admin                          -0.055244   0.038026  -1.453
presenting_complaintGenitourinary                                    -0.236477   0.034159  -6.923
presenting_complaintGenitourinary >> Unable to pass urine            -0.205932   0.044768  -4.600
presenting_complaintHead and neck                                    -0.726214   0.053349 -13.612
presenting_complaintNeurological                                      0.035823   0.039793   0.900
presenting_complaintNeurological >> Confusion                         0.004858   0.038502   0.126
presenting_complaintNeurological >> Dizziness                        -0.650210   0.042758 -15.207
presenting_complaintNeurological >> Falls / unsteady on feet         -0.067100   0.027676  -2.424
presenting_complaintNeurological >> Headache                         -0.670723   0.047047 -14.257
presenting_complaintNeurological >> Limb weakness                    -0.144926   0.049602  -2.922
presenting_complaintNeurological >> Speech disturbance               -0.131529   0.055585  -2.366
presenting_complaintObGyn                                            -0.242751   0.130138  -1.865
```

```
presenting_complaintOther                                          -0.592582   0.052671 -11.251
presenting_complaintPsychosocial / Behaviour change                -0.804196   0.062775 -12.811
presenting_complaintSkin                                           -0.532667   0.066844  -7.969
presenting_complaintSkin >> Localised swelling / redness / lumps / bumps -0.427720   0.047806  -8.947
presenting_complaintTrauma / musculoskeletal                       -0.418605   0.032410 -12.916
presenting_complaintTrauma / musculoskeletal >> Head injury        -0.423749   0.038746 -10.937
presenting_complaintTrauma / musculoskeletal >> Injury of lower limb     -0.341395   0.048680  -7.013
presenting_complaintTrauma / musculoskeletal >> Pain in lower limb       -0.320936   0.036282  -8.845
presenting_complaintUnwell Adult                                   -0.303797   0.068293  -4.448
```

In [354…

```
model_mega_scaled <- lmer(adjusted_score ~ female + age_scaled + news_scaled + imd_scaled + filab_scaled + PRUH + presenting_complaint + (1
summary(model_mega_scaled)
```

```
Correlation matrix not shown by default, as p = 39 > 12.
Use print(obj, correlation=TRUE)  or
    vcov(obj)        if you need it
```

```
Linear mixed model fit by REML ['lmerMod']
Formula: adjusted_score ~ female + age_scaled + news_scaled + imd_scaled +      filab_scaled + PRUH + presenting_complaint + (1 | patient_Tr
ustNumber) +      (1 | rater)
   Data: smaller_multi

REML criterion at convergence: 103152.8

Scaled residuals:
    Min      1Q  Median      3Q     Max
-3.8286 -0.5832  0.0019  0.5847  5.1409

Random effects:
 Groups              Name        Variance Std.Dev.
 patient_TrustNumber (Intercept) 0.9585   0.9790
 rater               (Intercept) 0.4460   0.6678
 Residual                        1.4942   1.2224
Number of obs: 28535, groups:  patient_TrustNumber, 11085; rater, 381

Fixed effects:
                                                                    Estimate Std. Error t value
(Intercept)                                                         4.533261   0.059548  76.128
femaleTRUE                                                          0.087670   0.025302   3.465
age_scaled                                                          0.520451   0.013084  39.778
news_scaled                                                         0.114601   0.009231  12.414
imd_scaled                                                         -0.165144   0.015624 -10.570
filab_scaled                                                        0.151972   0.010076  15.082
PRUHTRUE                                                           -0.778338   0.109075  -7.136
presenting_complaintAirway / breathing                             -0.067568   0.174669  -0.387
presenting_complaintAirway / breathing >> Difficulty breathing     -0.181956   0.077315  -2.353
presenting_complaintAirway / breathing >> Short of breath          -0.346780   0.043167  -8.033
presenting_complaintCirculation / chest                            -0.200992   0.082945  -2.423
presenting_complaintCirculation / chest >> Chest pain              -0.611019   0.044511 -13.728
presenting_complaintCirculation / chest >> Collapse / fainting episode   -0.421282   0.074920  -5.623
presenting_complaintCirculation / chest >> Palpitations            -0.695003   0.079783  -8.711
presenting_complaintEnvironmental                                  -0.460351   0.202250  -2.276
presenting_complaintEye                                            -0.605942   0.149906  -4.042
presenting_complaintGastrointestinal                               -0.198711   0.048660  -4.084
presenting_complaintGastrointestinal >> Abdominal pain             -0.366868   0.045344  -8.091
presenting_complaintGeneral / minor / admin                        -0.099583   0.057270  -1.739
presenting_complaintGenitourinary                                  -0.191119   0.049711  -3.845
presenting_complaintGenitourinary >> Unable to pass urine          -0.116916   0.066118  -1.768
presenting_complaintHead and neck                                  -0.493658   0.087130  -5.666
presenting_complaintNeurological                                    0.059003   0.062858   0.939
presenting_complaintNeurological >> Confusion                       0.071972   0.058215   1.236
presenting_complaintNeurological >> Dizziness                      -0.540088   0.061836  -8.734
presenting_complaintNeurological >> Falls / unsteady on feet        0.056042   0.042776   1.310
```

```
presenting_complaintNeurological >> Headache                          -0.509687   0.070368  -7.243
presenting_complaintNeurological >> Limb weakness                     -0.201658   0.082378  -2.448
presenting_complaintNeurological >> Speech disturbance                -0.202251   0.096026  -2.106
presenting_complaintObGyn                                             -0.002182   0.201632  -0.011
presenting_complaintOther                                             -0.644975   0.077665  -8.305
presenting_complaintPsychosocial / Behaviour change                   -0.560380   0.097373  -5.755
presenting_complaintSkin                                              -0.348974   0.115350  -3.025
presenting_complaintSkin >> Localised swelling / redness / lumps / bumps -0.240994   0.071851  -3.354
presenting_complaintTrauma / musculoskeletal                          -0.182013   0.052082  -3.495
presenting_complaintTrauma / musculoskeletal >> Head injury           -0.165903   0.061305  -2.706
presenting_complaintTrauma / musculoskeletal >> Injury of lower limb  -0.108228   0.080143  -1.350
presenting_complaintTrauma / musculoskeletal >> Pain in lower limb    -0.193199   0.056731  -3.405
presenting_complaintUnwell Adult                                      -0.528669   0.098813  -5.350
```

In [356…

```
patient_variability <- smaller_multi %>%
  group_by(patient_TrustNumber) %>%
  summarise(cfs_range = max(adjusted_score) - min(adjusted_score),
            visit_count = n())

# Look at patients with multiple visits
multi_visit_variability <- patient_variability %>%
  filter(visit_count > 1)

summary(multi_visit_variability$cfs_range)
```

```
   Min. 1st Qu.  Median    Mean 3rd Qu.    Max. 
  0.000   1.000   2.000   2.114   3.000   7.500 
```

In [420…

```
table(multi_visit_variability$cfs_range)
```

```
   0    1  1.5    2  2.5    3  3.5    4  4.5    5  5.5    6  6.5  7.5 
2798 2317 1082 1820 1347 1243 1124  595  911  138  474   21  134   40 
```

In [529…

```
model_mega <- lmer(adjusted_score ~ patient_GenderCode + age_scaled + NEWS_Score + filab + PRUH +
               (1 + filab + NEWS_Score + patient_GenderCode + age_scaled  | rater) +
               (1 + age_scaled | patient_TrustNumber),
               data = smaller_multi)
```

```
boundary (singular) fit: see help('isSingular')
```

In [531…

```
summary(model_mega)
performance::icc(model_mega)
```

```
Linear mixed model fit by REML ['lmerMod']
Formula: adjusted_score ~ patient_GenderCode + age_scaled + NEWS_Score +      filab + PRUH + (1 + filab + NEWS_Score + patient_GenderCode +
age_scaled | rater) + (1 + age_scaled | patient_TrustNumber)
   Data: smaller_multi

REML criterion at convergence: 106052.7

Scaled residuals:
    Min      1Q  Median      3Q     Max
-4.0644 -0.5687 -0.0042  0.5769  5.1482

Random effects:
 Groups              Name                  Variance Std.Dev. Corr
 patient_TrustNumber (Intercept)           1.028968 1.01438
                     age_scaled            0.005372 0.07329  -1.00
 rater               (Intercept)           0.571729 0.75613
                     filab                 0.431372 0.65679  -0.55
                     NEWS_Score            0.001867 0.04321  -0.05  0.33
                     patient_GenderCodeMale 0.001478 0.03844   0.49 -0.50 -0.89
                     age_scaled            0.020602 0.14353  -0.36  0.69  0.43 -0.51
 Residual                                  1.473977 1.21407
Number of obs: 29243, groups:  patient_TrustNumber, 11330; rater, 381

Fixed effects:
                        Estimate Std. Error t value
(Intercept)             3.820129   0.065547  58.281
patient_GenderCodeMale -0.079407   0.025575  -3.105
age_scaled              0.550477   0.017221  31.966
NEWS_Score              0.081270   0.007762  10.470
filab                   1.468606   0.106305  13.815
PRUHTRUE               -0.980414   0.108311  -9.052

Correlation of Fixed Effects:
            (Intr) pt_GCM ag_scl NEWS_S filab
ptnt_GndrCM -0.099
age_scaled  -0.188  0.031
NEWS_Score  -0.202 -0.075  0.148
filab       -0.631 -0.070  0.195  0.011
PRUHTRUE    -0.362 -0.001 -0.011  0.006  0.056
optimizer (nloptwrap) convergence code: 0 (OK)
boundary (singular) fit: see help('isSingular')
Warning message:
“Can't compute random effect variances. Some variance components equal zero. Your model may suffer from singularity (see `?lme4::isSingular`
and `?performance::check_singularity`).
  Solution: Respecify random structure! You may also decrease the `tolerance` level to enforce the calculation of random effect variances.”
```

<NA>

In [598…

```
summary(model_mega)
```

```
Linear mixed model fit by REML ['lmerMod']
Formula: adjusted_score ~ patient_GenderCode + age_scaled + NEWS_Score +      filab + PRUH + (1 + filab + NEWS_Score + patient_GenderCode +
age_scaled | rater) + (1 + age_scaled | patient_TrustNumber)
   Data: smaller_multi

REML criterion at convergence: 106052.7

Scaled residuals:
    Min      1Q  Median      3Q     Max
-4.0644 -0.5687 -0.0042  0.5769  5.1482

Random effects:
 Groups              Name                   Variance Std.Dev. Corr
 patient_TrustNumber (Intercept)            1.028968 1.01438
                     age_scaled             0.005372 0.07329  -1.00
 rater               (Intercept)            0.571729 0.75613
                     filab                  0.431372 0.65679  -0.55
                     NEWS_Score             0.001867 0.04321  -0.05  0.33
                     patient_GenderCodeMale 0.001478 0.03844   0.49 -0.50 -0.89
                     age_scaled             0.020602 0.14353  -0.36  0.69  0.43 -0.51
 Residual                                   1.473977 1.21407
Number of obs: 29243, groups:  patient_TrustNumber, 11330; rater, 381

Fixed effects:
                        Estimate Std. Error t value
(Intercept)             3.820129   0.065547  58.281
patient_GenderCodeMale -0.079407   0.025575  -3.105
age_scaled              0.550477   0.017221  31.966
NEWS_Score              0.081270   0.007762  10.470
filab                   1.468606   0.106305  13.815
PRUHTRUE               -0.980414   0.108311  -9.052

Correlation of Fixed Effects:
            (Intr) pt_GCM ag_scl NEWS_S filab
ptnt_GndrCM -0.099
age_scaled  -0.188  0.031
NEWS_Score  -0.202 -0.075  0.148
filab       -0.631 -0.070  0.195  0.011
PRUHTRUE    -0.362 -0.001 -0.011  0.006  0.056
optimizer (nloptwrap) convergence code: 0 (OK)
boundary (singular) fit: see help('isSingular')
```

In [260…

```r
# Create season variable
smaller_multi$season <- factor(quarter(smaller_multi$DateTime, with_year = FALSE),
                               labels = c("Winter", "Spring", "Summer", "Autumn"))
# Add day of week to the dataset
smaller_multi$day_of_week <- wday(smaller_multi$DateTime, label = TRUE)


# Calculate the estimated marginal means for day of week
library(emmeans)

# Create time of day variable
smaller_multi$time_of_day <- factor(case_when(
  hour(smaller_multi$DateTime) >= 0 & hour(smaller_multi$DateTime) < 6 ~ "Night",
  hour(smaller_multi$DateTime) >= 6 & hour(smaller_multi$DateTime) < 12 ~ "Morning",
  hour(smaller_multi$DateTime) >= 12 & hour(smaller_multi$DateTime) < 18 ~ "Afternoon",
  hour(smaller_multi$DateTime) >= 18 & hour(smaller_multi$DateTime) < 24 ~ "Evening"
))

# Update your existing models to include season and time of day

# Model with filab
model_filab_temporal <- lmer(adjusted_score ~
                             patient_GenderCode + age_scaled + filab +
                             season + time_of_day + day_of_week +
                             (1 + age_scaled | patient_TrustNumber) +
                             (1 | rater),
                             data = smaller_multi)

# Model with filab interaction and random slope
model_filab_interaction_random_slope_temporal <- lmer(adjusted_score ~
                                                      patient_GenderCode + age_scaled * filab +
                                                      season + time_of_day +
                                                      (1 + filab | rater) +
                                                      (1 + age_scaled | patient_TrustNumber),
                                                      data = smaller_multi)

# Vanilla model
model_vanilla_temporal <- lmer(adjusted_score ~
                               patient_GenderCode + age_scaled +
                               season + time_of_day +
                               (1 | rater) +
                               (1 + age_scaled | patient_TrustNumber),
                               data = smaller_multi)

# Model with NEWS and filab
model_news_and_fi_temporal <- lmer(adjusted_score ~
```

```
                                    patient_GenderCode + age_scaled + NEWS_Score + filab +
                                    season + time_of_day + day_of_week +
                                    (1 + filab | rater) +
                                    (1 + age_scaled | patient_TrustNumber),
                                    data = smaller_multi)

# # Compare models
# anova(model_filab, model_filab_temporal)
# anova(model_filab_interaction_random_slope, model_filab_interaction_random_slope_temporal)
# anova(model_vanilla, model_vanilla_temporal)
# anova(model_news_and_fi, model_news_and_fi_temporal)
```

```
boundary (singular) fit: see help('isSingular')

Warning message in checkConv(attr(opt, "derivs"), opt$par, ctrl = control$checkConv, :
“Model failed to converge with max|grad| = 0.00460642 (tol = 0.002, component 1)”
boundary (singular) fit: see help('isSingular')

boundary (singular) fit: see help('isSingular')
```

In [261…

```
# If you want to see the specific effects of season and time of day in any model
library(emmeans)
summary(emmeans(model_news_and_fi_temporal, specs = ~ season))
summary(emmeans(model_news_and_fi_temporal, specs = ~ time_of_day))
summary(emmeans(model_news_and_fi_temporal, specs = ~ day_of_week))
```

```
Note: D.f. calculations have been disabled because the number of observations exceeds 3000.
To enable adjustments, add the argument 'pbkrtest.limit = 29243' (or larger)
[or, globally, 'set emm_options(pbkrtest.limit = 29243)' or larger];
but be warned that this may result in large computation time and memory use.

Note: D.f. calculations have been disabled because the number of observations exceeds 3000.
To enable adjustments, add the argument 'lmerTest.limit = 29243' (or larger)
[or, globally, 'set emm_options(lmerTest.limit = 29243)' or larger];
but be warned that this may result in large computation time and memory use.
```

A summary_emm: 4 × 6

	season	emmean	SE	df	asymp.LCL	asymp.UCL
	<fct>	<dbl>	<dbl>	<dbl>	<dbl>	<dbl>
**1**	Winter	4.144355	0.05161089	Inf	4.043200	4.245511
**2**	Spring	4.211306	0.05177264	Inf	4.109833	4.312778
**3**	Summer	4.269464	0.05107923	Inf	4.169350	4.369577
**4**	Autumn	4.266664	0.05100086	Inf	4.166704	4.366624

```
Note: D.f. calculations have been disabled because the number of observations exceeds 3000.
To enable adjustments, add the argument 'pbkrtest.limit = 29243' (or larger)
[or, globally, 'set emm_options(pbkrtest.limit = 29243)' or larger];
but be warned that this may result in large computation time and memory use.

Note: D.f. calculations have been disabled because the number of observations exceeds 3000.
To enable adjustments, add the argument 'lmerTest.limit = 29243' (or larger)
[or, globally, 'set emm_options(lmerTest.limit = 29243)' or larger];
but be warned that this may result in large computation time and memory use.
```

A summary_emm: 4 × 6

	time_of_day	emmean	SE	df	asymp.LCL	asymp.UCL
	<fct>	<dbl>	<dbl>	<dbl>	<dbl>	<dbl>
**1**	Afternoon	4.231184	0.04983902	Inf	4.133502	4.328867
**2**	Evening	4.245717	0.05115323	Inf	4.145459	4.345976
**3**	Morning	4.153422	0.05112592	Inf	4.053217	4.253627
**4**	Night	4.261465	0.05568893	Inf	4.152317	4.370613

```
Note: D.f. calculations have been disabled because the number of observations exceeds 3000.
To enable adjustments, add the argument 'pbkrtest.limit = 29243' (or larger)
[or, globally, 'set emm_options(pbkrtest.limit = 29243)' or larger];
but be warned that this may result in large computation time and memory use.

Note: D.f. calculations have been disabled because the number of observations exceeds 3000.
To enable adjustments, add the argument 'lmerTest.limit = 29243' (or larger)
[or, globally, 'set emm_options(lmerTest.limit = 29243)' or larger];
but be warned that this may result in large computation time and memory use.
```

A summary_emm: 7 × 6

	day_of_week	emmean	SE	df	asymp.LCL	asymp.UCL
	<fct>	<dbl>	<dbl>	<dbl>	<dbl>	<dbl>
1	Sun	4.248238	0.05446661	Inf	4.141486	4.354991
2	Mon	4.213810	0.05302279	Inf	4.109888	4.317733
3	Tue	4.239401	0.05292183	Inf	4.135676	4.343126
4	Wed	4.248002	0.05290135	Inf	4.144317	4.351687
5	Thu	4.205704	0.05303264	Inf	4.101761	4.309646
6	Fri	4.208177	0.05304657	Inf	4.104207	4.312146
7	Sat	4.197298	0.05419577	Inf	4.091077	4.303520
